# Systems Leadership: a qualitative systematic review of advice for policymakers

**DOI:** 10.12688/openreseurope.18982.1

**Published:** 2025-01-10

**Authors:** Paul Cairney, Claire Toomey

**Affiliations:** 1 Division of History, Heritage, and Politics, University of Stirling, Stirling, Scotland, FK94LA, UK

**Keywords:** policy, policymaking, governance, systems leadership, complexity theory, wicked problems

## Abstract

**Background:**

‘Systems leadership’ research rejects the idea that complex policy problems can be solved by a few heroic leaders in the centre of government or at the top of organisations. Many people need to contribute to systems leadership by collaborating to harness the skills of actors across government and outside of government. At times, this proposition is vague and it is difficult to know who should change or what to do. We searched the academic and grey literature for further advice on how to foster effective systems leadership.

**Methods:**

We conducted a qualitative systematic review (2024) of peer reviewed journal articles (Web of Science) and grey literature reports (Policy Commons, Overton). Each text had to inform advice on systems leadership. We used an immersive inductive approach to identify key sources of ideas, highlight common themes, and relate the results to insights from policy theories.

**Results:**

84 texts met the inclusion criteria (39 Web of Science, 34 Policy Commons, 11 Overton), then we included 80 snowballed academic and grey references (total 164). Some relate leadership to complexity theory, but most provide broad accounts of systems leadership that emphasise decentralisation and collaboration. These accounts describe high aspirations and essential skills but limited evidence from activity. Nevertheless, this literature helps us produce a coherent synthesis of common insights and advice on how to foster systems leadership.

**Conclusions:**

We identify key features of systems leadership: reject heroic top-down leadership and central control in favour of collaboration across boundaries; develop attributes (e.g. humility), mindsets (e.g. big picture), and skills (e.g. facilitation) to act in complex systems (albeit without a common view on what a complex system is); and, seek organisational and political support for this approach. Some accounts identify barriers to systems leadership and negative experiences, while others redefine such obstacles as learning opportunities.

## Plain language summary

‘Systems leadership’ describes collective action towards a common goal: harnessing the energy of many, and spanning boundaries across policy sectors or outside government. There is not – and should not be – a single centre of authority. Old ideas of heroic leaders are not useful when facing new reality. Policy problems are ‘wicked’. Leadership takes place in a complex policymaking system where outcomes emerge in the absence of central control, prompting collaboration to face uncertainty and ambiguity. Systems leadership requires attributes such as humility, and new mindsets and skills to foster collaboration.

This description is more aspirational and conceptual than empirical. Most describe why there should be systems leadership. Some describe its advantages (such as more effective or equitable policymaking) and what key elements would look like. Many highlight a lack of applications and the difficulty of evaluating success.

There is debate about how to describe the role and influence of systems leaders: to be humble about the limits to their influence (the new mindset), or to show their disproportionate value (to demonstrate their benefits to sceptical audiences).

There is doubt about the novelty of systems leadership. One story is of radically new ways to understand the world, to reject reductionist approaches to science and rationalist approaches to policymaking. Another is of connections to other collaborative approaches emphasising adaptability to fluid contexts and making sure that stakeholders and citizens are involved.

Part of these doubts come from the vagueness of concepts such as leadership, complex systems, mindsets, and collaboration. ‘Systems leadership’ can mean too much and therefore too little. Its ambiguity has initial value – to bring in actors with similar aims and values – but carries the potential for disenchantment if actors find few clear benefits. Systems leaderships enthusiasts offer tangible ways to address this challenge and reframe it as an opportunity.

## Introduction

We present a qualitative systematic review of advice for systems leadership. This review is the second part of a series on collaborative policymaking ( Cairney & Toomey, 2024a). It extends our search for usable evidence to inform advice on how to improve policymaking, while recognising that the idea of more effective policymaking is vague and contested. The core starting point for each review is the frequent rejection by policy scholars of ‘an old story of elite policy analysis and centralised authority’ in favour of collaboration (2024a: 4). The new story argues that:

- A ‘single centre of government to coordinate policymaking’ is not *possible*, given the limits to the coordinative resources of policymakers, or *desirable*, at least in liberal democracies seeking to distribute powers and responsibilities.
- Policy problems are too complex to be amenable to simple agreed-upon solutions. They ‘do not respect traditional government boundaries, and require collaborative responses across government and between governmental and non-governmental actors’
- If policymaking responsibilities are distributed across political systems, we need new ways to improve ‘collaboration to foster policy coherence and policymaking integration’
- Our response to this dilemma needs to recognise two reference points: aspirational stories of how policymaking should work, and research-informed stories of policymaking reality (2024a: 8–10, drawing on Cairney, 2024a).

We draw on academic and grey literatures to seek advice from scholars and practitioners on how to connect systems leadership to this agenda. We relate systems leadership advice to stories of ‘how people want policymaking to work, and how it really works’ ( Cairney & Toomey, 2024a: 4). Aspirational stories relate leadership to requirements: to work together to improve government and democracy. Policy theory-informed studies focus on how policymaking works, to identify a gap between requirement and reality, and provoke discussion of how to respond. Combined, these stories create different demands on new advice, to inform aspiration and realistic assessment.

We described our first search for advice - on collaborative policymaking - as akin to seeking a needle in a haystack, since too few scholars and practitioners define this term far less give concrete advice. There are more grounds for optimism with ‘systems leadership’ since scholars engage frequently and thoughtfully with its meaning, albeit while not defining leadership or producing a common definition of complex systems. Most definitional work begins by describing what systems leadership does *not* mean, to signal a shift from old conceptions of charismatic individuals. It challenges a top-down model of ‘hero leadership’ ( Mangan & Lawrence-Pietroni, 2019) that: praises exceptional people at the top of organisations, describes the need for strong leaders to make hard decisions authoritatively and for their followers to carry them out, and expects to find a clear line between decisions from the top and outcomes at the bottom ( Avolio *et al.*, 2009). In its place is a focus on collaboration between many actors and organisations, to reflect the complex nature of policy problems (and policymaking processes) that are not solvable by one leader or organisation and require collective action ‘among different organizations, sectors, and even countries’ ( Senge *et al.*, 2015). This narrative returns us to the aspiration of ‘collaborative policymaking’ then introduces concepts ‘such as collaborative, distributed, dispersed, collective and systems leadership’ that might help ( Mangan & Lawrence-Pietroni, 2019: 83).

In that context, we continue our search for ‘pragmatic advice that offers hope for individual and collective responses but tempered by engagement in a system over which they do not have full understanding or control’ ( Cairney & Toomey, 2024a: 4). We continue to ‘cast our net as widely as possible, to search for any potentially relevant advice’, including ‘aspirational advice’ on what good systems leadership might look like, and ‘pragmatic and context-specific accounts of what worked, for whom, and in what context’. If a study used the phrase ‘systems leadership’, and offered or informed advice, we reviewed its findings. Further, since there is clearly a collection of terms that inform similar ideas, we snowball from included texts to explore connected approaches (including complexity leadership or complex systems leadership) and the extent to which they share the same origin story or reference points.

We summarise the wide range of advice sheltering under the umbrella term ‘systems leadership’ (Results) then synthesise these findings to produce themes relevant to collaborative policymaking (Discussion). As with the first review, we find no support for a blueprint, not least because the emphasis is on agile and flexible leadership ( Mangan & Lawrence-Pietroni, 2019), which makes ‘the search for a uniform how-to-guide for collaborative policymaking … a fool’s errand’ ( Cairney & Toomey, 2024a: 5). Still, it makes sense to identify a small number of practices associated with positive stories of systems leadership, including the need to collaborate across boundaries, develop attributes, mindsets, and skills to act in complex systems and seek organisational and political support for this approach.

## Methods

Our aim is to produce connected reviews using a consistent method of qualitative systematic review. As such, the following description draws heavily on Cairney and Toomey (2024a), which adapts Kuckertz and Block’s (2021) guidance on describing systematic reviews then describes our response to key limitations.

*Rationale*. We seek to understand advice on how to improve policymaking with reference to systems leadership, as part of a wider focus on effective collaboration. We present the second review for the Horizon Europe project *Healthy Working environments for all Ages: An evidence-driven framework* (WAge). WAge’s Work Package 1 seeks to support collaborative policymaking to foster the uptake of usable evidence, which requires us to understand how collaborative policy processes or initiatives work. Further, our WP1 explores the idea that policy may change from the ‘top down’ or ‘bottom up’, and systems leadership informs both dynamics.

*Engagement with previous reviews*. Cairney leads a team conducting a series of qualitative systematic reviews that connect key aims or strategies to policy theory insights ( Cairney *et al.*, 2021; Cairney & Kippin, 2022; Cairney *et al.*, 2023; Cairney & Toomey, 2024a). This is the fifth review submitted to Open Research Europe, but the second to focus on collaboration and seek advice from academic and grey sources.

*Research/guiding questions*. Our most general guiding question was: What advice do scholars and practitioners offer on systems leadership? We used these sub-questions to guide our initial search and reflection:

1. How many studies use the term ‘systems leadership’ to describe their object of study?
2. How do they describe systems leadership? For example, do authors describe their pursuit of better leadership and how to do it, or study efforts in organisations relevant to policy (e.g. governments)?
3. What factors do they describe as constraining or facilitating systems leadership?
4. In what context does this pursuit of systems leadership take place? For example, within one government or across multiple levels? Across policy sectors? To foster collaboration between government and non-governmental actors?
5. What findings or recommendations do they provide?
6. To what extent are these lessons transferable to other contexts?

We anticipated that each included text would define systems leadership (to some extent) then offer at least one of three kinds of advice:

1. Aspirational: what would good systems leadership look like?
2. General: what context-free advice do people give to foster systems leadership?
3. Context-specific: what works, for whom, and in what context, and what lessons are transferable to other contexts? While the wording is resonant with realist review, we did not expect to find enough information to review in that way (based on our previous experiences).

*Databases and initial search terms*. We began by searching for advice from one main database of peer reviewed academic texts (Web of Science, WOS) and two of the grey literature, including government, non-governmental organisation, academic and think tank reports (comparing Policy Commons, PC and Overton). We used the search term ‘systems leadership’, anticipating that it would (1) maximise coverage among texts using this term and require a high level of manual searching (which was inevitable, given our need to search manually for advice), and (2) provide partial coverage of relevant work using different terminology. We address the latter with an unusually high amount of snowballing (almost half of our total texts) to seek key sources for this field and identify some strongly connected terms (e.g. complexity leadership) while considering separate reviews for other terms (e.g. see Aoki *et al.*, 2024 on whole of government approaches). We learned from our previous review that the addition of multiple academic databases would have rapidly decreasing returns compared to snowballing. Our initial experience with WOS and PC/Overton suggested that further broad searches would be a poor use of resources, and we found that snowballing helped to identify key academic (e.g. Senge *et al.*, 2015) and grey sources (e.g. Ghate *et al.*, 2013a; Ghate *et al.*, 2013b).

*Timeliness*. We ran searches up to 10 July 2024 (WOS), 1 ^st^ July (PC) and 8th July (Overton).

*Manual searches and choices regarding initial inclusion*. We used similar criteria for inclusion as our other four reviews, including publication in English and not restricting initial inclusion in relation to research method (we focused more on relevance). Our preference was to set an initially low inclusion bar to be able to immerse ourselves in the literature, identify the relevance of texts, and generate a narrative of the field to help to make sense of advice. We followed Cairney and Toomey (2024a) to remove the need for a grey literature report to engage with policy concepts to be included (previous reviews examined levels of conceptual engagement).

**Figure 1.**
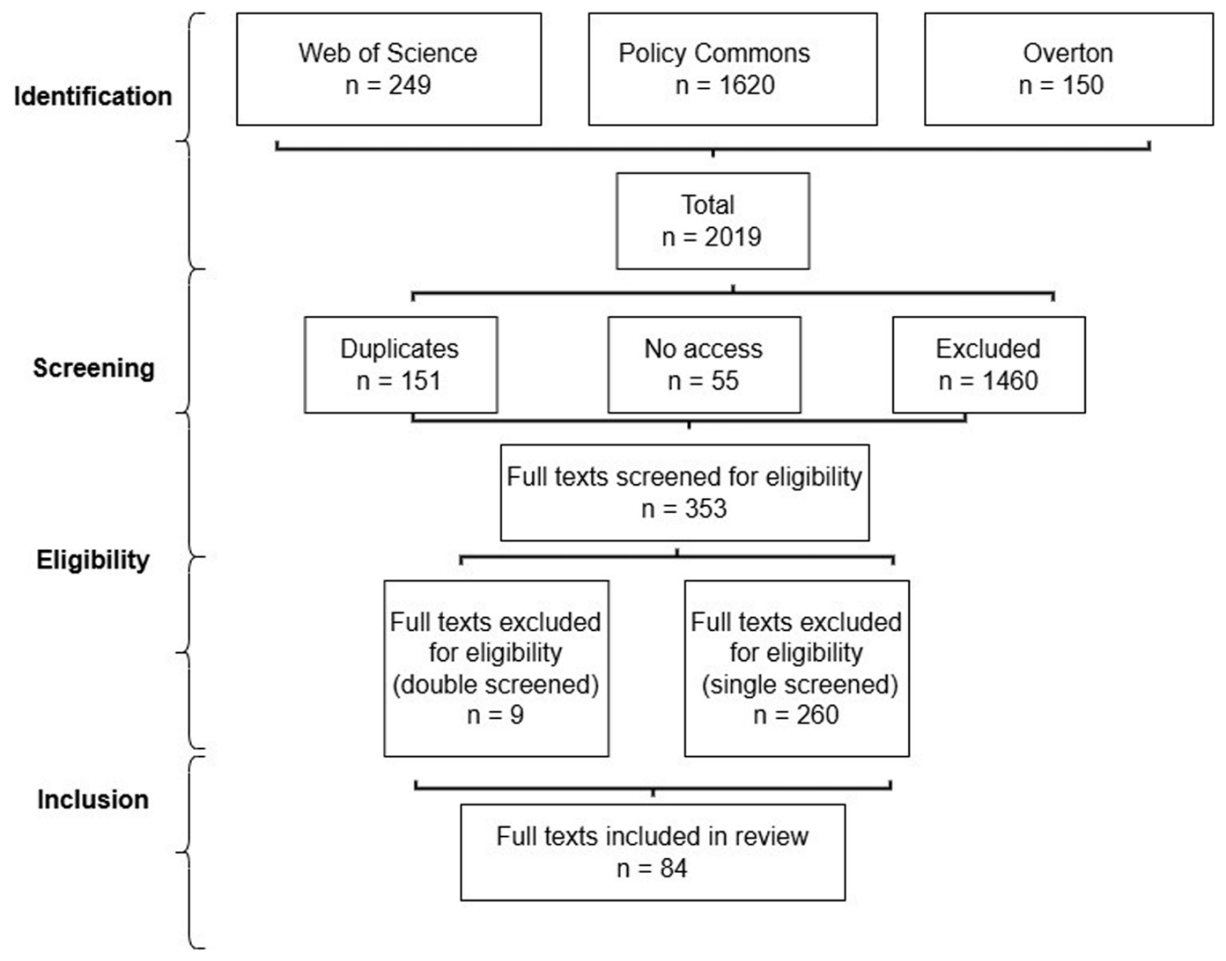
Review process flow chart.

Toomey conducted a manual search of each text in a two-stage process to include texts that: (1) referred to the phrase ‘systems leadership’ (or close equivalent, such as system or complexity leadership) in the title, abstract, and/ or introduction, and (2) offered advice in the main text (or information to support advice, such as if describing the effectiveness of collaboration strategies) ( Table 1 and Figure 1).

**Table 1.** Search results 2024.

Database	Search results	Duplicates	No access	Excluded	Included
Web of Science	249	7	29	174	39
Policy Commons	1620	139	11	1436	34
Overton	150	5	15	119	11
Grand Total	2019	151	55	1729	84

*Routine data collection*. Toomey coded the following aspects (Excel):

- *Country/region of study*. Publication in English skews the results to authors in Global North countries focusing on that country. Countries like the UK, US, Canada, and New Zealand command an unusually high proportion of entries. The WOS search skewed largely to UK (9/39) and US (8) and Canada (2), accounting for 19/39 or 49% (the other 20/39 had no notable pattern). PC skewed to Canada (12/34), New Zealand (8), UK (6), US (4), Australia (2), which accounted for 32/34 or 94% (plus one European Commission and 1 Spain). Overton was UK (8/11, 73%) plus 1 Canada, UN, and World Economic Forum.
- *Sectors or issues*. Some specific issues are discussed frequently: health and social care integration in England (academic, government agency, and think tank publications), and education improvement in Canada (the numbers are misleading because Ontario’s Institute for Educational Leadership published many small documents). Beyond these examples, most studies related generally to issues such as the need for new forms of training, professional practice, advice for leaders, or organisational changes to support systems leadership (which made precise % coding difficult).
- *Methods or approaches*. WOS articles (39) included qualitative interview (10), case study (8), discussion or conceptual analysis (7), literature review (5), mixed methods (4) and miscellaneous approaches (e.g. describing online training or simulation). Too few PC or Overton studies describe methods to justify further analysis here (although many think tank reports offer new data based on elite interview).
- *Definitions*. Too few articles provide a clear definition of systems leadership to justify further analysis here.

*Data analysis, aggregation, and presentation*. As with previous reviews, Cairney used an inductive qualitative approach to analyse each text, generate themes manually (Results), and synthesise advice from the results (Results and Discussion). As each previous review notes, ‘the rules associated with this method are less prescriptive than with its quantitative equivalent, suggesting that we (a) describe each key judgement, and (b) foster respect for each authors methods and aims’ ( Cairney & Kippin, 2022, citing Sandelowski & Barroso, 2007: xv). We did not perform any quantitative tests to assess risk of bias. We present a narrative systematic review (rather than qualitative coding aided by tests for inter-coder reliability).

### Limitations relevant to data gathering and analysis

The nature of our task *and limitations to our search* influence our approach to data analysis and presentation. We identified five limitations in our first review ( Cairney & Toomey, 2024a: 7), which we use to identify fewer limitations with the current review. First, although few authors define systems leadership, it is not used as loosely as ‘collaborative policymaking’. We were able to synthesise these texts to provide a broad definition with commonly described elements (Results). The more relevant limitation relates to our focus on *systems* leadership which highlights other leadership approaches (e.g. distributed) but without presenting a full review. Second, while many studies shelter under the same umbrella term, the systems leadership literature is less disparate, and authors show how it relates to other leadership terms. Third, the systems leadership literature is more likely to offer advice, such as to recommend new training for leadership skills. Fourth, while our grey literature searches produced a low ratio of effort to reward, our larger snowballing activity helped to reveal a useful common reference to key grey texts (the 80 snowballed texts was almost as large as the 84 texts included from searches). Further, the PC/Overton process helped to reach a sense of saturation (after WOS snowballing, few new OC or Overton texts gave us additional information or insights). Fifth, while much research identifies what we don’t know, a key message from systems leadership scholars is to embrace such uncertainty. Overall, the available research is better matched to our task than in our first review.

As with the first review, we respond in three ways: keeping the bar for inclusion low to encourage immersion in the field; snowballing to identify from key reference points, primarily by following up studies described in included texts as important sources (plus some texts from expert recommendations); and, making it explicit when we present our synthesis of insights and offer our interpretation of practical advice. This immersive approach threw up some curveballs (e.g. Williams, 2021) or texts that engage sparingly with the ‘systems leadership’ lexicon (e.g. Chow, 2013), but in the context of a fairly coherent collection of discussions that could be usefully synthesised.

Indeed, we were struck by high levels of awareness between academic and grey literatures, in general – for example, when multiple academic studies cite Ghate *et al.* (2013a) or grey reports cite Senge *et al.* (2015) - and in fields like UK healthcare and public health studies where think tank reports are key sources for (and draw on) academic work (e.g. Timmins, 2015). The snowballing process also helped to give us some more confidence in the grey literature searches, such as when they both highlighted the same reports or collections. There are also overlaps between academic and grey literatures in these databases (e.g. Overton lists Swanwick & McKimm, 2011).

The complete search protocol, PRISMA checklist, and structured bibliography is stored on Open Science Framework (OSF) https://osf.io/hr769/

## Results

### What is systems leadership?

Almost no included study defines ‘leadership’, partly because it defies common understanding. Indeed, some are sceptical about its usefulness for research ( Hazy *et al.*, 2007: 3; Silva, 2016). The exception is Dickson (2009: 295), who compares with a manager/administrator to define a leader as an actor who ‘goes first’ and ‘takes on the responsibility of acting to shape the future’ (see also Senge *et al.* (2015: 28), ‘to step across a threshold’, and Welbourn *et al.* (2012: 4) on management as ‘control’ and leadership as ‘influence’).

There is no single definition of systems leadership. However, we can identify frequently similar reference points or arguments, such as to: explain what systems leadership is *not,* to signal a shift in thinking; identify the need to respond more effectively to complexity; and, emphasise the value of new leadership approaches. This negative definition is more useful than it appears, since it allows us to identify a common origin story on which to build a definition: *old approaches focused too much on great leaders, and new approaches shift our focus to leadership; they highlight the need to respond to wicked problems and policymaking complexity, and suggest that systems leadership may be more effective and democratic than top-down or hierarchical approaches.* We summarise this argument in Table 2 then explore each element.

**Table 2.** Defining systems leadership.

What systems leadership is *not*	Reject a fixation with heroic individual leaders and top-down leadership. There is not – and should not be – a single centre of policymaking authority.
What systems leadership is	Foster collective action towards a common goal or vision. Harness the energy and skills of many actors. Span boundaries across policy sectors, government departments, levels of government, or outside government
Addressing wicked problems	Collaborate to address problems that defy technical definitions and simple solutions.
Navigating policymaking complexity	Navigate a complex policymaking system where high uncertainty and ambiguity are features not bugs, and outcomes emerge in the absence of central control.
The advantages of systems leadership	Systems leadership is good for democracy and effectiveness. Doing the right thing promises big rewards.
Systems leadership is part of a collection of new approaches	Systems leadership is part of a wider conceptual and normative movement to reject old approaches to leaders and hierarchy.

***Old approaches to leadership focused too much on great leaders. New approaches shift our focus from leaders to leadership***

Old studies emphasised personal qualities such as charisma and decisiveness. Effective leadership was hierarchical, policy was made from the top, and leaders had high influence on their followers (expected to carry out their aims):

‘Our traditional views of leaders – as special people who set the direction, make the individual decisions, make the key decisions, and energize the troops – are deeply routed in an individualistic and nonsystemic worldview. Especially in the West, leaders are *heroes* – great men (and occasionally women) who “rise to the fore” in times of crisis’ ( Senge, 1990: 340; see also Bertini, 2019: 10 on the legacy ‘image of a senior UN official’ as an ‘older man’ from the ‘North’).

New approaches shift from heroic leaders to ‘the collective and relational dynamics of *leadership* practice’ ( Bolden *et al.*, 2020: 26). They identify the new attributes, mindsets, and skills required of many actors spread across a system, and shift from rationalist top-down approaches towards adapting to systems out of their control ( Uhl-Bien *et al.*, 2007: 299; 302). They accentuate a wider organisational and policymaking dynamic in which many actors contribute to leadership, such as to design and deliver a collective vision. These actors depend on each other to get things done, in systems characterised by the distribution of power and limited potential for central control ( Avolio *et al.*, 2009; Dickson, 2009: 295; Osborn *et al.*, 2002: 799). For example:

‘accounts of effective systems leaders are about as distant from the idea of the traditional ‘hero leader’ - fearless, forceful and uncompromising - as it is possible to get … all about the skilful harnessing and holding in creative tension the energy in the wider system, rather than driving through change by sheer force of will and exercise of power … as frequently about ‘willingness to give things away’ as … achievement of one’s own goals or promoting of one’s own agency agenda’ ( Ghate *et al.*, 2013a: 8).

This focus on what systems leadership is *not* sets up a binary between top-down/hierarchical versus systems leadership ( Lichtenstein & Plowman, 2009: 618; Onyx & Leonard, 2011: 496). Examples include:

- From (1) ‘commanding, telling, persuading, influencing, motivating - conceived as activities in which there is a point of origin (leaders) and a point of reception (followers)’, to (2) ‘dialogue and sense-making - conceived as … mutually constituted social achievements’ ( Drath *et al.*, 2008: 639; 651).
- From ‘prescriptive’ to ‘adaptive’, ‘individual organisations’ to ‘place-based’ and ‘whole system’, ‘centralised’ to ‘distributed participatory’ and ‘co-productive’, regulatory and policymaker accountability to ‘people and communities’, short to long-term, ‘competitive’ to ‘consensus-seeking’ (requiring us to surface rather than ‘avoid conflict’) ( Edmonstone, 2020: 357).
- From a ‘competitive’ to ‘collaborative mindset’, an internal organisational to a systemic focus, management skills (e.g. ‘planning, organizing’) to leadership skills (e.g. inspiration, visioning, change facilitation’), and, ‘top-down’ to more inclusive decision-making ( Dickson, 2009: 300).
- Replace ‘formal, managerial, institutional, competitive’ approaches that are ‘reliant on experts and specialists’ with new values ‘embodied in the development of an informal network marked by self-organization, collaboration, agility, wide participation and transparency’ ( Taylor *et al.*, 2020: 56).
- From ‘a linear and narrow approach’ to ‘a complex adaptive arrangement spanning across organizations and institutions’ ( Blankart *et al.*, 2024: 2).
- Develop teams rather than ‘just individuals’ and ‘leadership across systems of care rather than just institutions, and on followership as well as leadership’ ( The King’s Fund, 2011; see also Anandaciva *et al.*, 2018: 55).
- Reject an ‘over-simplistic model of change management’ (in education) which ‘pays insufficient attention to the complexities of the policy process’ and ‘casts teachers in the role of passive implementers of policy, in the process de-professionalising teachers at all levels of the system whilst holding them accountable’ ( Mowat, 2019: 65).
- A role “not necessarily to lead people ‘over the top’ to a utopian future” but rather ‘to disappoint people at a rate they can manage’ ( Grint, 2020: 2).
- Replace heroic individual scientists (in the West or Global North) by developing local adaptive capacity among ‘academic biomedical researchers in Africa’ ( Rose *et al.*, 2024: 10).

One partial exception is Bertini’s (2019: 23) description of ‘Desirable qualities of a UN leader’, combining systems leader qualities (e.g. focusing on trust, self-awareness, listening, empowering staff) with traditional notions of courage, decisiveness, ‘Making difficult decisions’, and ‘maintaining a “toughness in leadership”’ (see also Club de Madrid & Edelman, 2021: 10–13; Onyx & Leonard, 2011: 503–6).

***New approaches reflect the rise of wicked policy problems***

The world’s most pressing problems are ‘wicked’: a term coined by Rittel and Webber (1973) and still used frequently as a shorthand for problems that defy rationalist policy analysis. Wicked problems resist simple technical definitions, and their size, severity and cause are contested. They defy simple solutions: it is difficult to know if all possible solutions have been identified or what is working, and trial-and-error learning is controversial since any policy produces major social and economic effects (1973: 161–67, summarised in Cairney & Kippin, 2024: 155). While Rittel and Webber (1973) used ‘wicked’ to compare with the ‘tame’ issues more amenable to technical definition and simple solutions, and these terms took off in academic and practitioner studies, they were never properly defined, and it is tempting to write off any tricky political problem as wicked ( Turnbull & Hoppe, 2019). Further, Auld *et al.* (2021: 127–8) have ramped up this language to describe ‘super wicked problems’ in which ‘time is running out’, key actors are responsible for exacerbating *and* solving the problem, there is ‘no central authority’, and actors put too much value on the short-term costs of action at the expense of long-term benefits.

Nevertheless, ‘wicked’ serves a rhetorical purpose to note that, if policy problems are not easily defined or amenable to simple solutions, then old forms of leadership are ill-equipped to deal with them, necessitating the ‘new ways of thinking, working and collaboration’ ( Bolden, 2020; Evans *et al.*, 2021; Hunter, 2009; Taylor *et al.*, 2020: 51). Accounts may describe wicked problems as our new reality, or relate them to a new crises, such as when the combination of reduced funding and higher demand for public services prompts the search for systems change:

‘Wicked challenges are complex, intractable, ambiguous, dynamic and open-ended. They cannot be solved with analysis or optimization. Instead, stakeholders must collaborate to define the problem and to find a mutually acceptable resolution. Wicked problems require negotiating values and identities’ ( Talley & Hull, 2023: 1040).
‘Leaders talk of wrestling with persistent ‘wicked issues’ that shape-shift and resist resolution, and which cannot be solved by single agencies acting alone. They also talk of ‘a one-time opportunity’ to make changes that can streamline services sufficiently to withstand the turbulence and ensure their survival and even improvement into the future’ ( Ghate *et al.*, 2013a: 5).

Examples of problems requiring new leadership include: the Sustainable Development Goals (SDGs), ‘each representing complex systems – such as climate, food, health, cities – with myriad stakeholders’ ( Dreier *et al.*, 2019: 4), obesity, sustainable development ( Bigland *et al.*, 2020: 2), ‘food systems change’ ( Enderton *et al.*, 2017: 94; O’Malley *et al.*, 2022; Public Policy Forum and CAPI, 2017), health systems and vaccination programmes in light of Covid-19 ( Beharrell *et al.*, 2021: 93; Gulati & Busari, 2022), water contamination management ( Grey *et al.*, 2023), social and economic inequalities ( Taylor *et al.*, 2020: 51); homelessness ( Jang *et al.*, 2016); road safety ( US Department of Transportation (2016); regeneration after natural disaster ( Department of the Prime Minister and Cabinet, 2017); and emergency management ( Caro, 2016: 114; Club de Madrid and Edelman, 2021).

***New approaches reflect the increasing complexity of policymaking or governance in which there is no single centre of authority or control***

Complexity theory has *some* influence on systems leadership research, but many accounts do not explain this connection (they use citation delegation). Further, many define complex systems in different ways or with reference to different systems. For example, to relate systems leadership to our wider focus on policymaking, we begin by describing complex *policymaking* systems and how its features can relate to a lexicon including *non-linearity*, *sensitivity to initial conditions*, *self-organisation*, and *emergence* (see also Nel & Taeihagh, 2024):

1. “A complex policymaking system is greater than the sum of its parts those parts are interdependent – elements interact with each other, share information and combine to produce systemic behaviour.
2. Some attempts to influence complex systems are dampened (negative feedback) while others are ampliﬁed (positive feedback). Small actions can have large effects and large actions can have small effects.
3. Complex systems are particularly sensitive to initial conditions that produce a long-term momentum or ‘path dependence’.
4. They exhibit ‘emergence’, or behaviour that results from the interaction between elements at a local level rather than central direction.
5. They may contain ‘strange attractors’ or demonstrate extended regularities of behaviour which may be interrupted by short bursts of change” ( Cairney, 2013, summarising Cairney, 2012: 348).

Such terms need translation to each context. For example, in political science, path dependence’ resonates with studies of historical institutionalism, in which choices and rules created in the distant past still influence current practices ( Cairney, 2012: 350). Further, ‘punctuated equilibrium theory’ uses the language of feedback to describe ‘macropolitical’ policymaker attention lurching from issue to issue, contributing to a small number of major policy changes when attention is high and large number of minor changes when low ( Baumgartner *et al.,* 2023). ‘Self-organization’ also resonates with governance and implementation in which there may be ‘self-organizing networks’ ( Rhodes, 1997: 50) or ‘self-selecting clusters of organisations in which a variety of public and private organisations cooperate’ ( Hjern & Porter, 1981, cited in Cairney, 2012: 351), contributing to a sense that policy outcomes ‘emerge’ in the absence of central government control.

Compared to other complex systems (e.g. systems biology), it makes more sense in politics and policymaking to describe a centre of government with unclear influence on a system rather than the absence of a centre ( Cairney & Geyer, 2015). This point is essential whenever we seek to:

1. explain attempts to translate complexity theory into advice for policymakers, such as to encourage long-term trial-and-error learning at local levels rather than maintaining high stakes and short-term centralised performance targets ( Geyer, 2012), while
2. recognising that such advice is hard to reconcile with the language of democratic accountability (especially in Westminster political systems – Cairney *et al.*, 2019).

Then, we can relate such ideas to different scales of policymaking activity, such as when policy problems transcend jurisdictional boundaries and there is an absence of one authoritative government ( Auld *et al.*, 2021).

The systems leadership literature is not as easy to sum-up, for three relevant reasons (see also Jackson, 2024: 32 and Cairney, 2024b). First, ‘systems leadership’ is an umbrella term under which many approaches seek shelter (compare with the vagueness of ‘systems thinking’, Carey *et al.*, 2015: 3). In the included texts, we find a large set that capture a general sense of organisational, governance, or policymaking *complexity,* and a small subset of authors that relate systems leadership to properties of *complex systems* (often preferring terms such as ‘complexity leadership’, Uhl-Bien *et al.*, 2007). Within the latter, scholars tend to pick their own nomenclature and origin story to inform leadership research. Lichtenstein and Plowman (2009: 626; 620) cite Lichtenstein (2007) on ‘14 complexity theories ... used to explore leadership’, while noting their own unusual preference to relate ‘complex adaptive systems theory’ to the work of Prigogine on chemical systems (compare with Dreier *et al.*, 2019: 9). This variety is a wider feature of approaches to complex systems ( Nel & Taeihagh, 2024). The technical language of complex systems is abstract, informed by different key authors or institutes in multiple disciplines, and amenable to multiple interpretations and translations, prompting leadership scholars to make sense of key terms in different ways.

For example, while for us ‘emergence’ can refer to policy outcomes that result from local rule development in policymaking systems, to others it can describe something bigger, such as the ‘emergence’ of ‘novel, “higher” level structures, patterns, processes, properties, dynamics, and laws’ ( Hazy *et al.*, 2007: 6) or to ‘new forms of social cooperation in social networks’ ( Goldstein *et al.*, 2010: 13). In other words, emergence may describe *systems change*. If so, we may all explore the emergence of phenomena that ‘seem to have a “life of their own”’ (2010: 13) but not what phenomena to expect (or desire). A similar point can be made of the philosophy of complex systems, which can be described as if they exist independent of our experience of them, or with reference to how actors perceive and respond to them in different ways ( Jackson, 2024: 22).

Second, in systems leadership studies, the focus of study varies, such as on individual non-governmental organisations or a governance network comprised of many governmental and non-governmental organisations. We may be zooming in to analyse particular systems, or zooming out to identify systems of systems, without knowing how to separate such foci analytically. We need to recognise different conceptualisations of ‘networks’, ‘markets’, ‘collaborations’, and ‘social movements’ and encourage people to clarify what they mean by ‘whole systems’ approaches ( Welbourn *et al.*, 2012: 20). While Ospina (2017: 275–6) makes a convincing case to challenge the artificial separation between leadership studies in public and private sectors - not least because non-governmental organisations are increasingly a part of modern governance – this conceptual uncertainty matters.

Third, some focus on *system* leadership (singular), such as when addressing mental health ( Deputy Prime Minister and Minister for Health, 2021), transport ( Ministry of Transport, 2019), land information ( Land Information New Zealand, 2015), schools ( The Institute for Educational Leadership, 2014; The Institute for Educational Leadership, 2016; The Institute for Educational Leadership, 2017; The Institute for Educational Leadership, 2020), high performance sport ( AIS, 2021), or a professional scientific role within a government department ( Aston & Sweetland, 2024). The singular term can reflect country-specific usage, to refer to one specific system and omit the language of complex systems, such as when New Zealand government documents are describing a system of government agencies and partners to join-up.

Nevertheless, we can still identify useful broad themes throughout multiple accounts, such as in summaries of ‘complexity leadership’ in organisations:

‘leadership is viewed as an interactive system of dynamic, unpredictable agents that interact with each other in complex feedback networks, which can then produce adaptive outcomes such as knowledge dissemination, learning, innovation, and further adaptation to change … to achieve optimal performance, organizations cannot be designed with simple, rationalized structures that underestimate the complexity of the context in which the organization must function and adapt … Simply viewing the leader and follower in a simple exchange process won’t fly in terms of explaining the full dynamics of leadership’ ( Avolio *et al.*, 2009: 430)
‘Complexity Leadership Theory, a leadership paradigm that focuses on enabling the learning, creative, and adaptive capacity of complex adaptive systems (CAS) within a context of knowledge-producing organizations. This conceptual framework includes three entangled leadership roles (i.e., adaptive leadership, administrative leadership, and enabling leadership) that reflect a dynamic relationship between the bureaucratic, administrative functions of the organization and the emergent, informal dynamics of complex adaptive systems (CAS)’ ( Uhl-Bien *et al.*, 2007: 298).

Further, when expanding analysis from organisations to policy processes, we can identify similar reference points for a story of wicked policy problems and policymaking complexity, such as by using the shorthand of ‘volatile, uncertain, complex and ambiguous (VUCA)’ ( Bigland *et al.*, 2020: 2; Bolden *et al.*, 2023: 339; Cady & Heykoop, 2024: 129; Jackson, 2024: 21; Education Scotland, 2019; Elliott *et al.*, 2020: 202; Mangan & Lawrence-Pietroni, 2019: 83; Mangan & Pietroni, 2021: 1455; see also Osborn *et al.*, 2002 on ‘stability, crisis, dynamic equilibrium, and edge of chaos’, and Caro, 2016: 116 on embracing ‘uncertainty, non-linearity and emergent behaviours’). This reference point may explain a new social and economic context for private companies - ‘driven by globalization, technology, deregulation, and democratization’ ( Uhl-Bien *et al.*, 2007: 300) – *and* connect systems leadership to public and private policy and policymaking ( Goldstein *et al.*, 2010; Jackson, 2024; Uhl-Bien & Arena, 2017; Wheatley, 1999).

In that context, we can relate complexity to practicality, such as to explore how to respond (and what training to seek):

‘Systems Leadership describes the way people need to behave when they face large, complex and seemingly intractable problems; where they need to juggle multiple uncertainties; where no one person or organisation can find the solution on their own; where everyone is grappling with how to make resources meet growing demand; and where the way forward lies in involving as many people’s energies, ideas, talents and expertise as possible’ ( Edmonstone, 2020: 357; The Leadership Centre, 2015: 1).

Then, we can seek connections to narratives of changing *policymaking* (our preferred umbrella term) or *governance*, a term often preferred by scholars to note the limits to a focus on govern *ment* when so many actors inside and outside of government make and influence policy ( Rhodes, 1997). For example, new leadership relates to a new context of public management, in which scholars and practitioners are searching for ways to transition from:

1. The now-old ‘New Public Management’ (NPM) model, emphasising clear lines of accountability via leaders managing performance in a hierarchy, towards
2. More collaborative governance, emphasising wider collective action, shared responsibility, and the need to relate governance to democratic values such as stakeholder engagement and citizen participation as well as some moves towards decentralisation ( Bolden *et al.*, 2020: 27 on ‘New Public Service’; Osborne, 2006 on New Public Governance; see also Cairney & Toomey, 2024a).

While many accounts do not hang their argument on the peg of complex systems, they still describe new forms of governance requiring new forms of leadership:

‘The New Public Service and New Public Governance paradigms emphasize the relationship between public servants and citizens … collaborative forms of governance, such as networks for knowledge, information sharing, service delivery, and policy reform, and multistakeholder groups as well as formal multisector partnerships for collective problem solving. … the heroic leader in policy contexts gives way to collective forms in which leaders from all sectors share the goal of making life better for communities’ ( Ospina, 2017: 276–7)

Many relate these ideas to key reference points in public administration, such as Moore’s (1995) ‘strategic triangle’ which lauds public managers pursuing ‘public value’ (see also O’Flynn, 2007). For example, Bryson *et al.* (2017: 641) describe a ‘new world’ that is a ‘polycentric, multi-nodal, multisector, multi-level, multi-actor, multi-logic, multi-media, multi-practice place characterized by complexity, dynamism, uncertainty and ambiguity in which a wide range of actors are engaged in public value creation and do so in shifting configurations” (also cited in Bolden *et al.*, 2020: 27; Bolden *et al.*, 2023: 338). This world is characterized by ambiguity and contestation to translate values and beliefs into policy, in a complex system in which outcomes emerge in the absence of central control, prompting systems leaders to develop new skills to facilitate collaboration and navigate policy and policymaking complexity ( Bolden *et al.*, 2020: 28).

The main theme, binding such approaches together, is that there is no single centre of power, authority, or control (some also argue that there *should not be* central control). Rather, in complex policymaking systems, outcomes emerge despite attempts by national central governments to exert control or, when we extend our focus beyond national jurisdictions, in the absence of one centre (see Cairney *et al.*, 2019 on multi-centric policymaking). Top-down control and hierarchical leadership becomes inappropriate or ineffective or both ( Edmonstone, 2020: 357; Marion & Uhl-Bien, 2001: 396; Onyx & Leonard, 2011: 496, citing Lichtenstein *et al.*, 2006). Variations on this theme include systems leadership:

- Within an organisation, when leadership does not relate solely to ‘line management responsibility’ ( Timmins, 2015: 7).
- In multi-organisational systems where ‘no single organisation can control the outcomes’ ( Bigland *et al.*, 2020: 1).
- When designing systems and arguing that ‘approaches that involve diverse community-based organizations are more likely to be effective than those pursued by a single organization’ ( Enderton *et al.*, 2017: 86).
- When seeking ‘coordination, collaboration and change in complex multi-stakeholder environments’ ( Bolden *et al.*, 2020: 26).
- When fostering an ‘authorising environment’ conducive to collaboration, such as by avoiding the prioritisation of your organisation’s goals, not relying on ‘positional authority’, and encouraging ‘challenge’ to the status quo ( Ghate *et al.*, 2013a: 11).
- When addressing problems that transcend jurisdictional boundaries and ‘no one has authority over all the stakeholders who must collaboratively construct a shared definition of the problem to be solved and the coordinated response to it’ ( Talley & Hull, 2023: 1041).
- When many policy instruments (including regulations) combine to produce a system over which ‘central authority is absent and cooperative relationships are required for progress’, prompting a search for alternatives to ‘top-down decision-making’ ( Grey *et al.*, 2023: 71; see also Cairney, 2024b).

***Systems leadership is more effective and democratic***

Policymakers in formal power are not the only actors essential to policy design and delivery ( Bolden *et al.*, 2020: 27), and systems leadership ‘extends individual leaders well beyond the usual limits of their formal responsibilities and authority’ ( Ghate *et al.*, 2013a: 5). It involves influencing others by drawing on leadership skills rather than only exerting influence through formal power (although people in positions of power are essential to this approach’s success). Claims for this leadership approach’s value include that it is more:

- *effective* at addressing wicked problems not amenable to simple solutions delivered from the top-down
- *efficient*, if public services find synergies
- *engaged* with stakeholders and communities, and
- *equitable*, when including diverse voices and ‘bringing marginalised groups into the design and delivery of services’ ( Bolden, 2020: 2; Stabler & Kempton, 2020: 1).

The case for effectiveness relates to responding to a new policymaking reality:

‘single agencies can no longer respond effectively to wicked issues unless they work collectively and across the system. Single agencies have neither the budget nor the human resources to respond to the current level of expectation and demand; nor do they have sufficient know-how for solving complex multi-dimensional problems unless they pool information and skills with others. This … is consistent with the now substantial evidence about what service users (and especially those with greatest needs) want and need from public services, which is not usually a ‘single service response’ … but a joined up, multi-dimensional response to a series of interlocking issues’ ( Ghate *et al.*, 2013a: 9).

This argument resonates among research interviewees seeking wider systems thinking and cross-organisational perspectives ( Evans *et al.*, 2021: 26). It has also taken off in contexts – such as health and social care integration in England – where many ‘local leaders’ seek ‘a cohesive approach to working together’ to co-create ‘clear, shared priorities that are grounded in the needs of their communities and not in the interests of individuals or their organisation’ ( NHS Confederation, 2014, cited in Bigland *et al.*, 2020: 2; see also Moore *et al.*, 2023: 364).

Some argue that systems leadership is *necessary* and *good*. The emphasis is on widening democracy (e.g. the diversity and equality of participation) and reaping the benefits. The right thing to do promises big rewards. This argument often comes from inspiring stories of systems change, facilitated by systems leaders, and recounted by trainers and observers. According to Senge *et al.*’s (2015: 28) influential account, systems leaders’ passionate commitment inspires commitment in others, their ability to see through another’s eyes prompts others to be ‘more open as well’, their willingness to listen and learn helps to build trust, then trust aids collaboration, people feel empowered to face uncertainty by asking questions and learning, and more ‘system leaders emerge’ to produce better outcomes:

‘situations previously suffering from polarization and inertia become more open, and what were previously seen as intractable problems become perceived as opportunities for innovation. Short-term reactive problem solving becomes more balanced with long-term value creation. And organizational self-interest becomes re-contextualized, as people discover that their and their organization’s success depends on creating well-being within the larger systems of which they are a part’ ( Senge *et al.*, 2015: 28).

***Systems leadership is part of a collection of new approaches***

A sub-set of systems leadership articles describe properties of complex systems, connecting leadership to other terms – including ‘systems thinking’ and ‘whole systems approaches’ – that emphasise the need to understand complex systems that are greater than the sum of their parts (e.g. Bigland *et al.*, 2020: 2; Lichtenstein *et al.*, 2006; Lichtenstein & Plowman, 2009; Lichtenstein, 2007; Mayanja *et al.*, 2021; Uhl-Bien & Arena, 2017) and require ‘non-linear and emergent leadership approaches’ (e.g. Edmonstone, 2020: 357; see also Meyers, 2024: 303; Enderton *et al.*, 2017: 86; Gupta *et al.*, 2023: 522 on the broad usage of terms like ‘systems change’ or ‘systemic change’; and Caro, 2016: 116 on ‘complex adaptive leadership’). ‘Emergent leadership’ may also help to describe ‘community development’ processes in complex social systems ( Onyx & Leonard, 2011: 493). In that context, Jackson (2024: 210) argues that such connections are too superficial unless complexity scholars make full use of available tools and insights from systems thinking (informing ‘critical systems leadership’).

Systems thinking may also connect to policy design, accompanied by models or techniques relevant to leadership (and collaborative policymaking – Cairney & Toomey, 2024a), including:

- ‘Boundary Critique’ to identify who should be supported in collaboration, and ‘Critical Systems Heuristics’ to explore who should benefit from a service and be included in design.
- ‘Strategic Choice Approach’, to ‘ *shape* people’s understandings of the multi-dimensional problem; *design* several packages of possible policy responses; *compare* these packages; and *choose* between them’.
- ‘Viable System Model’ to assess ‘the responsiveness of an organisation or multi-agency system to a changing world’.
- ‘Soft Systems Methodology’ to explore ‘different stakeholder perspectives’, and ‘Community Operational Research’ which ‘resists the top-down design and implementation of policy in favour of co-design and co-production with multiple stakeholders, communities and citizens’ ( Hobbs & Midgely, 2020: 2–3).

Most texts connect to a collection of leadership approaches that tell a similar origin story about the need to move on from heroic leaders and top-down approaches. The vagueness of leadership terms, and an incentive for academics and consultants to invent new descriptions, has prompted a plethora of terms that overlap with the general story of systems leadership, including ‘shared’, ‘collective’, ‘collaborative’, ‘plural’, ‘distributed’, ‘integrative’, ‘emergent’, ‘co’ and ‘democratic’ leadership” ( Ansell & Gash, 2012; Bolden, 2011: 251; Bolden *et al.*, 2020: 26; Bolden *et al.*, 2023: 339; Spillane, 2006). Systems leadership is the ‘close relative of adaptive leadership, and characterised by many of the same flexible, agile and nimble qualities needed by those leading complex organisations through change’ ( Ghate *et al.*, 2013a: 8; see also Lichtenstein & Plowman, 2009: 626). It may connect to ‘servant leadership’, which ‘is demonstrated by empowering and developing people; by expressing humility, authenticity, interpersonal acceptance, and stewardship’ ( Van Dierendonck, 2011: 1228). There are many accounts of these connections, including:

‘Theoretical strands in the collective leadership literature include network … complexity … discursive … and constructionist collective leadership … They share a view of leadership as an emergent, interactive process intended to cultivate group members’ capacity and adaptability to navigate complexity’ ( Ospina, 2017: 281).

Some relate their terminological preference to a stated aim. For example, Rissola and Haberleithner’s (2020) reference to *integrative leadership* reflects the European Commission’s pursuit of policy coherence and policymaking integration, supporting ‘entrepreneurs’ to connect government and business:

‘Integrative leadership is an emerging leadership approach that fosters collective action across many types of boundaries in order to achieve the common good. It brings together leadership concepts and practice rooted in five major sectors of society—business, government, nonprofits, media, and community. It focuses on leadership development at all levels, from individual to global’ ( Crosby, 2008, cited by Rissola & Haberleithner, 2020: 7).

Van Dyke (2013: 5) describes leadership focused on *sparking or managing systems change*, including ‘catalytic’ or ‘transformational’ leadership or ‘leadership of emergence’, often focused on place or community ( *place-based or civic leadership*) (compare with the ‘firestarter’ in ‘collaborative leadership’ for ‘deep systemic change’ in Dolezal, 2014: 2–4). Taylor *et al.* (2020: 50) describe overlaps between systems leadership ideas and a *social movement* for policy change (in the ‘2025 Movement’ to end avoidable health inequalities’ in North Wales by 2025). If *community engagement* describes one element of systems leadership, it connects to terms like ‘asset-based community development, participatory action research, local engagement, and public participation’ and invokes a range of levels of engagement described as ‘Inform, Consult, Involve, Collaborate, and Empower’ ( Enderton *et al.*, 2017: 86).

Rigby *et al.* (2019: 486) describe leadership with a value-based purpose: *equitable systems leadership.* It connects to terms – including ‘social justice leadership’, ‘distributed social justice leadership’, and ‘transformative leadership’- focused on combining ‘public activism’ and leadership to challenge racism and support marginalised populations in schools. The aim is to facilitate the leadership capacity of school staff, community members, and students and encourage self- and collective reflection on the inequitable practices maintained in school education (2019: 488; see also Gupta *et al.*, 2023 on ‘early intervention system leaders’ supporting early years children with disabilities). These roles bolster ‘shared and collaborative’ leadership capacity across professions and schools, ‘so that each school has multiple leaders to continually change practice’ ( Wratten, 2018: 9; 16).

There are multiple connections to a wider lexicon on leadership and values. A focus on rejecting top-down and inflexible managerialism, coupled with the need to humanise and empower people to work together – across traditional boundaries - to face uncertainty and complexity, connects to *creative bureaucracy* ( Landry & Caust, 2017). Inclusive collaboration for social system change connects to *transformative feminist leadership* ( Batliwala & Friedman, 2014). The focus on relationships, curiosity, and space for creativity, experimentation, and learning connects to ideas on the *moral imagination* for peace building and conflict resolution ( Lederach, 2005).

There are also connections to governance studies in which leadership plays a part, such as regarding ‘public value’ when the shift from NPM and search for more collaborative or decentralised governance is a common focus (e.g. Hartley *et al.*, 2019). In such cases, ‘systems leaders’ could perform roles in a system including: ‘entrepreneurs’ (providing challenge or new ideas), ‘insider-outsiders’ (seeking to bring in other actors), ‘commissioners’, ‘investors’ and ‘framework setters’ (allocating public and private resources to, and setting boundaries for, new ideas or practices), ‘historians’ (explaining how and why a system has come to this point), ‘visionaries’ (creating a vision for the future), ‘consumers’ (the early adopters, trying out new ideas), ‘scalers’ and ‘exiters’ (scaling up new and winding down old ideas), and ‘evaluators’ (seeking new ways to measure performance) ( Leadbeater & Winhall, 2020: 39–44 on systemic innovation).

Overall, we find that many approaches - including ‘systems leadership’ - tell a common origin story, emphasising the need to reject the old focus on heroic leaders and engage with wicked issues and policymaking complexity. This new story can promise more effective and/or more democratic approaches to leadership. From that origin, there is the choice of two paths. One seeks insights from complexity theory to situate leadership in complex systems. Another uses ‘systems’ loosely and connects to other leadership scholarship. Or, many leadership studies try to pursue both paths (if only to cite studies on complexity theory to contextualise their account).

### What is required for systems leadership?

Most accounts emphasise the strong connection between systems leadership, collective or collaborative action, and boundary spanning. There is a remarkably long list of required attributes, mindsets, and skills, prompting the need for more training and capacity, and for a policymaking environment or architecture conducive to systems leadership strategies. We summarise these requirements in Table 3.

**Table 3.** Requirements for systems leadership.

Collective action	Collaboration among policy actors across and outside of government
Boundary spanning	Working across traditional organisational, policy sector, professional, and disciplinary boundaries
General requirements	Principles and practices, roles, competencies, capacity
Relational attributes	Humility, reflection, self-awareness, supportiveness, empathy, compassion
Mindsets	Focused on the big picture Open-minded and collaborative Flexible, agile, curious, and open to learning Serving a moral purpose Ambition tempered by realism
Skills	Delegation, enabling, brokerage and linking, facilitating support and capacity towards systems change Evaluation Conflict resolution and political astuteness
A supportive environment or architecture for new strategies or a collective vision	Political support A supportive culture or climate Performance management support The time and space for development Training support A common focus, such as Place or Equity. Creative tension The accumulation of insights and learning

***Collective or collaborative action (inside and outside government)***

A key focus is on widespread collaboration across and inside/outside of government (compare with Cairney & Toomey, 2024a). This requirement may reflect a systemic context, characterised by interconnected or interdependent actors and organisations, and a democratic impetus, such as to generate stakeholder ownership and citizen participation ( Atkinson *et al.*, 2015: 9; Bolden *et al.*, 2023: 338; Evans *et al.*., 2021: 24, citing Ghate *et al.*, 2013a; Gulati & Busari, 2022). If so, collective leadership involves ‘a range of organisational and stakeholder cultures, often without direct managerial control of resources and working on issues of mutual concern that cannot be addressed by any one person or agency’ ( Edmonstone, 2020: 357). This approach should be reflected in accountability, where system actors take collective responsibility for collective action ( Beharrell *et al.*, 2021: 94). The aim is to support more democratic and collegial decision-making in the pursuit of improvement in a system ( Malby *et al.*, 2018: 794), relating leadership to ‘coalition-building’ in communities and complex systems ( Dreier *et al.*, 2019: 4). A collective approach requires ‘many people working at different levels and in different places, crossing boundaries’ ( Castelyn, 2024: 1).

***Boundary spanning***

Boundaries can be social, cultural, or professional, and relate to forms of knowledge production, geography, or levels or types of government ( O’Flynn, 2013: 13). Many boundaries have been constructed to serve a purpose (such as to assign resources to a well-defined organisation or aim) and endure regardless of efforts to cross them (2013: 13). Actors tell different stories about why they seek to cross boundaries, including to: foster coordination across organisations, manage the unintended consequences of creating boundaries (including policy incoherence or policymaking fragmentation), deal with complex problems that do not respect boundaries associated with government departments or policy sectors, foster ‘synergies’ between actors and organisations, and pool resources to work more effectively or produce better value (2013: 13–23). They may also pursue informal cooperation on an ad hoc or regular basis, and/or formalised coordination to ‘join up’ policy or create new networks subject to incentives and performance measures to oblige cooperation (2013: 23–36).

For example, included texts describe actors working in ‘complex, multi-player environments’ ( Gulati & Busari, 2022: 139), seeking ‘multicomponent, multisector, multisetting, and multilevel interventions’ ( Enderton *et al.*, 2017: 86), or performing leadership ‘across organizational and geopolitical boundaries, beyond individual professional disciplines, within a range of organizational and stakeholder cultures, and often without managerial control’ ( Van Dyke, 2013: 4). Many are trying to cut across boundaries between policy sectors (e.g. Public Policy Forum and CAPI, 2017: 6 on agri-food systems), geographies ( Edmonstone, 2020: 357), or ‘physical and virtual’ boundaries ( Evans *et al.*, 2021: 24, citing Ghate *et al.*, 2013a). As soon as they cross boundaries, many lose their ‘hierarchical authority to direct’, prompting them to ‘lead through influence, relationships and empowering others to catalyse change’ ( Grey *et al.*, 2023: 71). This process requires new perspectives, to understand ‘entire systems’ and work ‘in situations of deep complexity’ ( Talley & Hull, 2023: 1041).

***Essential attributes, mindsets, and skills***

We follow Mintrom ( 2019: 308, on ‘policy entrepreneurs’) to distinguish between *attributes* that ‘can be nurtured’ (e.g. ‘sociability’ and ‘tenacity’), *skills* that ‘can be learned’ (e.g. ‘team building’, ‘networking’), and *strategies* that rely on attributes/skills (e.g. ‘leading by example’, ‘scaling up change processes’). These elements are often hard to separate in overall descriptions of system leadership qualities, including:

‘leaders whose personal styles and attributes build strong relationships based on agreement around shared values; whose focus is on outcomes rather than compliance with processes; who can tolerate ambiguity and bring clarity to complex analyses; who understand that risk of failure inevitably accompanies experimentation and innovation; whose relentless curiosity and reflective style has led them to understand the perspectives of others; and who value the challenge that others bring to the table as strongly as they understand their own core objectives’ ( Ghate *et al.*, 2013a: 9).
‘individuals who can foster collective leadership in the face of systemic challenges in order to exceed the reach of existing institutions and their hierarchical structures of authority. … such leaders listen, empathise, cultivate networks of trust, instigate collaborations and pay particular attention to change at a larger scale’ ( Beharrell *et al.*, 2021: 93, citing Senge *et al.*, 2015).
‘systems leaders understand systems, encourage reflection, empathize with differing perspectives and prompt innovation. Their focus is on proactive problem-solving and value creation’ ( Talley & Hull, 2023: 1041).
Systems leaders need to face ‘a conflicting combination of constancy of purpose and flexibility’, foster a ‘coalition of the willing’, enjoy some stability in terms of the leadership team, include partners (such as citizens or stakeholders), consolidate the evidence for change, and ‘give away ownership’ and any credit for change ( Timmins, 2015: 8).

Still, a common starting point is to relate leadership to the context in which people interact. For example, Osborn *et al.* (2002: 800) argue that many approaches assume ‘stability’ when really we need to capture how to respond to ‘crisis’ involving ‘dramatic departure from prior practice’, ‘dynamic equilibrium’ when organisations are in ‘change mode’ to reflect new competition or technology, and the ‘edge of chaos’ or ‘transition zone delicately poised between order and chaos that many complex adaptive systems seem to naturally evolve toward’, requiring more collective and dynamic conceptions of leadership.

Then, studies relate crisis, dynamism, or transition to ‘personality traits’ and a ‘mindset’ which ‘nurtures a collegiate sense of problem solving’ ( Beharrell *et al.*, 2021: 96). Systems leaders need many relational attributes and skills to foster trust and collective action including: an open-mind, listening skills to facilitate joint understandings and reduce needless conflict across a system, resilience in the face of high stress and failure, flexibility rather than an attempt to assert authority, an investment of values and emotion into a joint vision, and the ability to deal with contradictory dynamics (e.g. when a system contains top-down and bottom-up elements) and uncertainty ( Bigland *et al.*, 2020: 7–12). These kinds of ‘systems thinking competencies’ help actors ‘to serve as catalysts for collective action leading to systems change .. working with diverse stakeholders in situations of informal and lateral authority’ ( Talley & Hull, 2023: 1041).

There are many useful attempts to distil these requirements into one coherent account of leadership. For example, Senge *et al.* (2015: 28–30) argue that systems leaders exhibit the energy, tenacity, and long-term commitment to develop three ‘core capabilities’:

1. *Perspective*. To see, and help others to see, the ‘larger system’ (e.g.to understand its ‘counterintuitive behaviour’ and potential to cause unintended consequences).
2. *Reflection*. To foster ‘reflection and more generative conversations’, which includes hearing other perspectives and ‘thinking about our thinking’ (to challenge our assumptions, be vulnerable, and be willing to ‘let go of pre-set goals and agendas’).
3. *Co-creation*. To shift the ‘collective focus from reactive problem solving to co-creating the future’ (e.g. helping people to face ‘difficult truths about the present reality’ and ‘inspire truly new approaches’).

Ghate *et al.* (2013a: 6–8) relate leadership qualities to ‘ways of …’:

- *feeling*, using personal values to drive motivation
- *perceiving* the big picture
- *hearing* diverse voices
- *relating* to many other essential actors
- *doing*, such as to stimulate discussion, facilitate cooperation, and enable others to take responsibility, and,
- *being*, from having ‘energy, drive and determination; bravery and resilience; confidence and the willingness to take risks’ to ‘humility and magnanimity’ and ‘patience’.

Their visualisation (2013a: 10; reproduced in Atkinson *et al.*, 2015: 3) of these ‘ways of …’ relates them to: (1) characteristics or aims of systems leadership, including ‘distributed’, ‘collective and participatory’, ‘shared power’, ‘relationship based’, influencing and nudging’, ‘shared vision and values’, ‘experimental and innovative’, and ‘disturbs the system’; and (2) the UK ‘public service context’, characterised by ‘decreasing resources’, ‘wicked issues’, ‘regulation and inspection’, ‘opportunity’, ‘paradox’, ‘interdependency and interconnectedness’, ‘risk’, and ‘VUCA’.

Jackson’s (2024: 210–12) list of requirements for ‘critical systems leadership’ (CST) are:

1. ‘Collective leadership and collaboration’ rather than heroic individual leaders.
2. ‘Communication of a vision and open dialogue’, using ‘softer skills … advocacy, shaping, influencing, enabling, consensus building and helping others live with uncertainty’
3. ‘Co-creation’ of that vision, by helping to assemble and empower coalitions of ‘willing stakeholders’.
4. ‘Managing the collaboration’, attending to the balance between diversity of opinion to spark innovation and open conflict that might undermine collaboration.
5. ‘An open approach to learning’, to question our own assumptions, listen, encourage challenge, and remain flexible in the face of uncertainty.
6. ‘An ethical orientation’, built on ‘honestly, integrity, transparency, humility, authenticity, compassion, understanding and self-awareness’.
7. ‘Promotion of appropriate evaluation’, seeking ways to monitor positive progress that are consistent with complexity theory insights (compare with Geyer, 2012; Ghate, 2016 on ‘whole system improvement’; Begg, 2020: 14: see also National Leadership Centre, 2020).

There is also a remarkably wide range of ways to describe essential requirements of systems leadership, from zooming into some attributes to zooming out to organise attributes, mindsets, and skills into an overall framework. The following list demonstrates our attempt to respect a variety of overlapping ways to describe systems leadership qualities.

**General requirements of systems leadership**

- *Principles and practices*. Uhl-Bien and Arena (2017) describe ‘five principles of enabling leadership (apply complexity thinking, enable adaptive space, leverage network structures, engage complexity dynamics, play in the pressures) and six practices of enabling leadership (brokerage, leveraging adaptive tension, linking up, tags and attractors, simple rules, network closure)’ (summary by Bolden *et al.*, 2020: 33).
- *Roles.* Ansell and Gash (2012: 2) describe collaborative leadership roles performed by external actors or emerging leaders from within: ‘ *Stewards* … helping to convene collaboration and maintain its integrity [before widespread ownership is possible]. *Mediators* … managing conflict and arbitrating exchange between stakeholders. *Catalysts* … helping to identify and realize value-creating opportunities [motivating many people to act]’.
- *Competencies.* Talley and Hull (2023: 1043–4) describe ‘competencies’ for leadership in complex systems, to enable a collective account of the direction of policy (skills in futures thinking, comparing the current and desired state), how to align future change with aims and objectives (strategic skills), and to foster collective commitment (facilitation skills through humility, sacrificing individual aims, fostering trust, and seeing things from multiple perspectives).
- *Capacity*. To foster systems leadership capacity across government, such as to ‘set the tone for an organisation to work more systemically and encourage staff to embed systems approaches in their work’, foster ‘cross-departmental and cross-directorate relationships so there are shared understanding of goals and of the system’, and use ‘systems principles and tools for complex problems’ (such as systems mapping) ( Government Office for Science, 2023: 2). Aston and Sweetland (2024: 22–3) describe ‘system leadership’ for Chief Science Advisers in UK government departments, shifting from an individualist role (‘being a pure adviser and technical expert’) towards managing a large staff to ‘elevate science’ across the department, and connecting to other CSAs across government.

Most accounts focus on the practices, roles, competencies, or capacity to deliver a vision for long-term systems change. Some try to squeeze such requirements into an acronym-based approach, such as the ‘CLEAR Framework for Leading Systems Change’: ‘convene and commit’ to foster stakeholder dialogue; ‘look and learn’, to generate collective understanding of a system; ‘engage and energize’ to ‘build trust, commitment, innovation and collaboration’; ‘act with accountability’ with reference to shared goals; and, ‘Review and revise’ regularly to reflect shifting contexts and the need for ‘an agile, flexible, innovative and learning-centered approach’ that welcomes ‘experimentation’ ( Dreier *et al.*, 2019: 4).

**Relational attributes of systems leaders**

- *Humility*. Stay humble, don’t seek credit, and try to see the world from other people’s eyes ( Timmins, 2015: 18). Motivate others with reference to shared values and vision, balance short and long term goals, exhibit curiosity, ‘embrace ambiguity’, ‘cultivate empathy’, and ‘play the role of an orchestra conductor … spotting and encouraging talent in a way that is harmonious and smooths out discordant notes’ ( Hagel & Mortensen, 2018: 9).
- *Reflection*. The ‘successful improvement leader’ is flexible, reflective, and willing to learn, and seeks ideas to incorporate ‘experiences and voice of staff, service users and the community’ ( Fawkes, 2013: 23).
- *Self-awareness*, including reflection on how your verbal and non-verbal communication combine ( Williams, 2021: 92). Avoid an ‘evangelical’ approach, in favour of fostering mutual learning and adapting to the routines and expectations of stakeholders ( Haynes *et al.*, 2020; also AIS, 2021 on ‘stakeholder management’)
- *Supportiveness and empathy* ( West *et al.*, 2017: 2)
- *Compassion*, to ‘be aware of, and seek to alleviate, the suffering of others’ ( Schroeder & Rowcliffe, 2019: 3; see also Cady & Heykoop, 2024: 131; Chow, 2013: 69)

*Compassionate systems leadership* emphasises ‘psychological safety and valuing diversity and positive attitudes to differences, to support a space where all voices can be heard’ and ‘creating space and freedom for individuals to experiment, discover and apply’ ( Koopmans *et al.*, 2022: 8). Requirements include ‘personal mastery (self-leadership)’ combined with ‘interpersonal skills (leading relationally), and systems thinking (connections between individuals, groups, and the wider community)’ ( Koopmans *et al.*, 2022: 3). Some compassionate roles relate to context, such as early years policies in Canada addressing ‘childhood vulnerability’, and a recognition that authors ‘learned from our Indigenous colleagues’ ( Schroeder & Rowcliffe, 2019: 3–6).

Similarly, *humanistic leadership* describes:

‘authentic leaders who are astute, empathic and possess cogent insight into human behaviours. Such leaders build relational and social capital upon which cogent collaboration is forged. Ethical behaviour, honesty and integrity are of paramount importance. Caring and compassionate leadership must prevail as an inspiration to all in the throes of emergencies. Courage and equanimity are also essential qualities of emergency leaders. Finally, humanistic leaders constantly challenge the status quo and strive for transformational changes in the best interests of the public’ ( Caro, 2016: 126)

Some actors may perform a ‘shepharding’ role to foster greater citizen participation in social systems or communities, such as to boost capacity when citizens have limited time and energy for participation, and guide citizens from broad interest to concerted action: ‘Shepherding is an intentional process of fostering trust, connecting … systems actors, tracking readiness, and making strategic requests to help interested community members define … system roles for themselves’ ( Enderton *et al.*, 2017: 85 on food systems).

**Mindsets of systems leaders**

- *Focused on the big picture*. A ‘systems thinking’ mindset focused on ‘wicked problems’ and a ‘holistic view’ informed by ‘multiple perspectives’ ( Nguyen *et al.*, 2023: 7). The ability to work with many ‘across whole systems’ ( Taylor *et al.*, 2020: 51). Making connections, having an open mindset, seeking multiple perspectives, being entrepreneurial and flexible in the face of uncertainty, fostering relationships, distributing leadership, and facilitating a ‘compelling vision which is shared by all partners in the whole system’ ( Welbourn *et al.*, 2012: 21). Connecting leadership to a wider focus on what is needed, including more flexible and participatory ways to design policy, stakeholder inclusion, trust building, learning, ‘local facilitation capacity, and new measures of systems change progress ( O’Malley *et al.*, 2022: 4)
- *Open-minded and collaborative*. Passionate and committed open-minded learners, curating conversations with stakeholders, challenging unequal power, and encouraging ‘innovation through ‘co-creation’ ( Dreier *et al.*, 2019: 35). Relating policy or subject expertise to a ‘collaborative culture’ that is inclusive of a ‘diverse groups of stakeholders’, being bold enough to challenge others, and being ‘agile and adaptive’ to anticipate and respond to change ( Jang *et al.*, 2016: 90).
- *Flexible, agile, curious, and open to learning*. Committed to continuous reflection and learning ( Beharrell *et al.*, 2021: 94). To remain flexible, comfortable with uncertainty, and learn from trial and error ( Begg, 2020: 10). To embrace paradoxes and tensions, such as when people make sense of problems in complementary or competing ways, prefer to work formally or informally, or seek to include or exclude certain actors ( Bolden *et al.*, 2023: 340).
- *Serving a moral purpose*. From the ‘great man’ focus on ‘charisma, intelligence, energy and dominance’ towards the ‘servant leader’ focus on ‘values, ethics and morals’ ( Swanwick & McKimm, 2011: 24, citing Greenleaf, 1970). Systems leaders exhibit ‘a clear moral purpose’, are role models, build networks and support others, set a ‘clear vision and direction’ and focus on ‘performance’ but not just in the short-term in one organisation ( Education Scotland, 2019).
- *Ambition tempered by realism*. Gupta *et al.* (2023: 529–36) describe the incongruence between new practices (to encourage systems change) and ‘business-as-usual’ or ‘how we do things’ when guiding practitioners, prompting new and reliable routines or procedures, ‘readying’ people for change through signposting and training, the maintenance of relationships across systems, and the provision of ‘systems interventions’ to ensure that changes are backed by financial and organisational resources.

For Jackson (2024: 212) a vague reference to ‘mindset’ – to see the whole system - is inadequate without methods to guide thinking. For example, the ‘Explore’ phase involves seeing a ‘situation of interest’ from five ‘systemic perspectives’: (1) ‘mechanical’ to clarify a goal and establish if necessary parts of a system help you to achieve it; (2) ‘interrelationships’ to anticipate systemic phenomenon such as ‘feedback loops’; (3) ‘organismic’, to treat one part of a system as the ‘brain’, able to learn from the operation of semi-autonomous elements; (4) ‘purposeful’, to accept that aspects of systems are socially constructed and be aware of social and political issues that may arise; and (5) ‘societal/environmental’, to foster democratic processes (e.g. are all voices heard, or some marginalised?) and ask if policies are sustainable (2024: 92–100).

**Skills for systems leadership**

- *Delegation*. To foster meaningful delegation to share responsibility, in the context of clear aims and instructions ( Williams, 2021: 92). To help colleagues use their empowerment but avoid ‘management anarchy’ associated with uncertainty about the bigger picture ( Senge & Sterman, 1992).
- *Enabling* skills, to project impartiality, avoid ego, observe, reflect, be inquisitive, empathetic, generous, and knowledgeable of multiple models and perspectives (to encourage ‘creativity’) ( Atkinson *et al.*, 2015: 11–13).
- *Brokerage* and *linking* to help people share ideas across networks, ‘leveraging adaptive tension’ to seek *some* conflict between ideas, and ‘tags and attractors’ that symbolize new ways of thinking, aided by ‘simple rules’ to guide such behaviour ( Uhl-Bien & Arena, 2017: 17).
- *Facilitation of support and capacity towards systems change*. Facilitated systems leadership – by an ‘enabler’ chosen for their place-based knowledge or positive reputation - generates: (1) a ‘shared sense of commitment’ to address the same wicked problem, ideally based on previous positive collaborations or backed by a written agreement to collaborate, (2) proper recognition of the need to change current routines, via problem-solving exercises to boost ‘experiential learning’ or direct engagement with citizens and stakeholders to challenge taken-for-granted perspectives, (3) a high and sustainable level of national and local level support for the policy agenda and change process (as well as the need to not prioritise more top-down processes), which (4) authorises less senior people to take forward collaborative work with stakeholders or take responsibility for action without facing blame ( Bolden *et al.*, 2020: 26–33).
- *Orchestration.* Reypens *et al.* (2021: 79) compare two approaches in networks: ‘dominating orchestration’ includes practices to design an initial proposal, assign roles, stimulate interaction, and connect stakeholders; ‘consensus-based orchestration’ involves supporting ‘bottom-up’ innovation, such as by motivating others, fostering smaller working groups, facilitating conversation, and monitoring progress.

Arnold and Wade (2017: 3–6) describe, ‘a complete set of systems thinking skills’ including to: see a whole system from multiple perspectives (some of which may challenge your initial beliefs); develop mental models, and reflect on how to act; make decisions in the face of uncertainty about the problem and effective of solutions; set boundaries around a system, and describe its properties accurately; identify and characterize relevant relationships; and understand past systemic behaviour (including feedback loops) to inform future action’ (see also Talley & Hull, 2023: 1040).

Further, The Policy Project (2015: 3–8) (New Zealand government) relates skills to roles, including: policy analysts and managers incentivised to gain experience across or outside of government, and empowered to take risks when innovating; the Head of the Policy Profession seeking a profession aligned to system-wide work; and, the role of ‘System Policy Capability’ to deal with ‘cross-cutting, systemic and complex issues’ ‘sharing talent’ and building trusting relationships across agencies (in the context of internal and external competition for staff). For example, the Inland Revenue (2021: 5) describes a process to help staff understand a shift of direction towards being more ‘customer-centric’ and creating larger and more flexible staff teams. Barker (2016: 4) describes a managed approach to ‘breaking out of team silos’ within government departments, such as by planning and resourcing a rotation of policy staff (culture and heritage policy).

We also identify the skill of *evaluation* in a field containing systems enthusiasts that may struggle to persuade their colleagues to take a leap of faith ( Jang *et al.*, 2016: 90). It is difficult to generate the ‘hard’ evidence required of investable policy reforms ( Bigland *et al.*, 2020: 11; Nguyen *et al.*, 2023: 8). Skills relate to making strategic or business cases while moving from top-down approaches associated with NPM performance management or implementation science (not to be confused with policy implementation studies – Nilsen *et al.*, 2013): ‘change the question from “how do I implement my evidence-based programme with the highest fidelity?” to “how can my evidence-based programme be implemented in a way that brings about systems change?”’ ( Begg, 2020: 14). This may require advocates to work with ‘a coalition of the willing’ to ‘build an evidence base’ ( Timmins, 2015: 13–17).

**Conflict resolution and political astuteness**

There is occasional reference to *conflict resolution* during difficult conversations on systems change ( Barkell & Snyder, 2021: 8 on ‘just culture’ approaches to patient safety). However, very few included studies relate leadership explicitly to political skills. Leaders need political astuteness when seeking change and relating political feasibility to, for example, the promises of a national governing party or beliefs of the multiple stakeholders necessary for collaboration. For example, when ‘whole systems’ approaches to reduce health inequalities - by addressing the ‘social determinants of health’ - falter, politicians may revert to individualist approaches with fewer political costs ( Hunter, 2009: 203). Here, political skills combine with tenacity to not lose enthusiasm following policy failure when dealing with ‘wicked issues’ ( Evans *et al.*, 2021: 27).

A collection of snowballed articles address the need to incorporate ‘micro politics’ or ‘organisational politics’ in leadership (in UK health and social care) ( Clarke *et al.*, 2021: 3). Many organisations – or actors or stakeholders within them – may exhibit different beliefs or preferences. If they use their power to get what they want, they may compete rather than collaborate in systems, especially if they have more resources than others ( Waring *et al.*, 2023a). Some may use their position or professional power to resist changes proposed by others ( Waring *et al.*, 2018: 2). Or, ‘street level bureaucrats’ may use their discretion to deliver policies in a way undermines collective aims ( Gale *et al.*, 2017: 9). The overall result could be a dispiriting sense of unfairness when actors perceive ‘winners and losers’ rather than the win-win aims of systems leadership ( Waring *et al.*, 2023a: 233).

In each case, the need to deal with the politics of systems change should be a key aspect of leadership training rather than wishing it away, pretending it does not exist, then learning from painful experience ( Waring *et al.*, 2018: 2–3; although Waring *et al.*, 2022; Waring *et al.*, 2023b show that professionals learn political skills from experience). This is important when ‘micro politics’ result from an unpredictable recurrence of bigger conflicts over values or grudges relating to perceptions of the power of other actors ( Waring *et al.*, 2023a: 241; compare with managing distrust in collaborative policymaking – Cairney & Toomey, 2024a: 18).

Political skills can relate to challenging unequal power dynamics by harnessing informal influence (e.g. to protect the role of each profession in health settings), working collectively as a profession (e.g. influencing national government policy), understanding ‘political dynamics’ to form alliances with useful actors ( Clarke *et al.*, 2021: 5–10), and ‘deal making’ to mitigate a sense among some actors that others were the bigger winners of collaboration ( Waring *et al.*, 2023a: 239). These skills supplement the collaborative skills emphasised in systems leadership, such as: ‘street level diplomacy’ to join-up initiatives at the point of delivery ( Gale *et al.*, 2017: 15); the role of clinical/medical directors in translating insights ‘between domains and forms of knowledge’, using diplomacy to anticipate and address levels of contestation that would not be recognised by non-specialists, and trying to ‘repair’ relationships after conflict (e.g. between hospital managers and clinicians) ( Jones & Fulop, 2021: 3–6; see also the overlap with ‘policy entrepreneur’ skills - Mintrom, 2019; Cairney, 2021).

***A supportive architecture and environment for new strategies***

We use ‘architecture’ to emphasise the scope to design support into strategies or approaches and ‘environment’ to describe conditions largely out of the control of participants (actors *adapt* to their wider environment). A supportive architecture for a collective vision for systems change may foster ‘direction, alignment and commitment’ (DAC) ( Talley & Hull, 2023: 1041). These terms map onto competences to promote a collectively agreed:

- *Direction*, to describe a positive future state and how to get there in cooperation with stakeholders (subject to competing beliefs)
- *Alignment*, to describe essential outcomes and milestones and anticipate obstacles (subject to trade-offs between objectives)
- *Commitment*, to consider ethical choices and unequal costs and benefits to action (subject to ‘overwhelming uncertainty of complex adaptive systems’) (2023: 1042, drawing on Drath *et al.*, 2008)

To that end, supportive elements can include:

*Political and performance management support*, to allocate resources to develop relationships, foster continuous training and support, and identify policy champions, backed by evaluations that value collaboration and long-term thinking ( Edmonstone, 2020: 359–61; Emmenegger *et al.*, 2019). To ‘build creativity and innovation into the appraisal and performance systems’ and avoid treating creative people as ‘mavericks’ in bureaucracies ( Bolden & O’Regan, 2018: 46–7).

*A supportive culture or climate.* West *et al.* (2017: 1) use the phrase ‘compassionate leadership’ to identify the ‘altruism’ and ‘intrinsic motivation’ of public service staff (in this case UK healthcare) and need for a culture conducive to systems thinking and leadership. It requires the shift from ‘a culture characterised by blame, fear and bullying’ to a:

‘culture of learning, where risk-taking (within safe boundaries) is encouraged and where there is an acceptance that not all innovation will be successful … staff feel confident in speaking out about errors, problems and uncertainties and feel empowered and supported to develop and implement ideas … They also work more co-operatively and collaboratively … in a climate characterised by cohesion, optimism and efficacy’ (2017: 2).

Similarly, Koopmans *et al.* (2022: 3, citing West *et al.*, 2017) describe requirements ‘for innovation to occur under compassionate leadership … (1) inspiring vision and strategy, (2) positive inclusion and participation, (3) enthusiastic team and cross boundary working, and (4) support and autonomy … [to] foster an environment that can provide space to think differently’. A key aim is to ‘foster inclusion, by acknowledging that individuals arrive with diversity in culture, education, experience, and discipline (professional background)’ ( Koopmans *et al.*, 2022: 8)

*The time and space for organic development*. Onyx and Leonard’s (2011: 503–6) synthesis of community leadership highlights ‘seven elements’ of ‘successful development’, including: leaders are ‘embedded’ in social networks but not initially in formal authority; decisions are shared at the ‘grass roots’ level; leaders recognise connections between multiple networks; they share a vision rooted in their knowledge of the community; they have practical management skills, including planning and organisation; there is succession planning; and they have clear ‘energy’ and ‘commitment’. Hulks *et al.* (2017: 5–11) relate the production of ‘a shared purpose and vision’ (such as in a strategic plan produced by multiple organisations) to the promise to work ‘together for the longer term’. This commitment may be bolstered by ‘frequent personal contact’ to build trust, ‘surface and resolve conflicts’ to anticipate issues before they cause major conflict, and ‘behave altruistically towards each other’, to avoid the unintended consequences of individualism or competition.

*Training support*. The case for reforms to leadership training have been made many times (e.g. Benington & Hartley, 2009 on the UK civil service) albeit with limited evidence of their roll out beyond specific case studies. For example, Beharrell *et al.*’s (2021: 94) case study interviewees had been identified following ‘training in leadership culture’.

*A common focus such as Place*. A focus on ‘place’ or a defined local area (or ‘whole area’) may foster a collective vision to be taken forward by actors or organisations with a clear and enduring role (e.g. UK Government ‘Total Place’; health and local government initiatives in England - Atkinson *et al.*, 2015; Goss, 2021; Local Government Association, 2018; Stabler & Kempton, 2020).

*A common focus such as Equity*. Acosta *et al.* (2021: 12–18) relate systems thinking to an ‘integrative resilience agenda’ that seeks to mainstream equity foci - in relation to initial context, the costs/benefits of action, and the rules of decision-making - in complex systems that can otherwise ‘reinforce persistent inequities if those systems are handled in sector-based silos.

*Creative tension*. Drivers of ‘adaptive leadership’ can include ‘collective identity formation’ when multiple actors interact, and ‘tension’ between ideas or when new information arrives ( Lichtenstein *et al.*, 2006: 5–6).

*The accumulation of insights and learning from long-term applications*. A supportive architecture and systems approach may become mutually reinforcing. For example, the language of systems and leadership is prevalent in aspects of Canadian education/ schools policy (albeit used generally with little mention of complexity theory). Here, the context is of school and student performance, where short-term goals for students matter, and the balance of accountability (e.g. between students, teachers, schools, and government) can be high stakes and contested (compare with Welsh Government, 2016). These ideas also matter to school district reforms exhibiting ‘growing pains’ and requiring meaningful communication with stakeholders ( The Institute for Educational Leadership, 2014: 1; 5). In that context, systems leadership combines with ideas about school improvement, individual motivation, and community engagement. ‘Collaborative professionalism’ and ‘coaching’ roles ( The Institute for Educational Leadership, 2019: 2; The Institute for Educational Leadership, 2020: 2–3) support leadership ‘driven by a process of collaboration and reciprocity’, which ‘can be exercised by different people (school leaders, system leaders, parents and teaching staff)’ ( The Institute for Educational Leadership, 2017: 3). Leaders should engage in ‘systems thinking’, including ‘futures thinking’ and ‘to understand the dense, complex and reciprocal connections among different elements of an organization’ ( The Institute for Educational Leadership, 2021a: 2). Individual attributes include ‘optimism’ (e.g. seeing good in others), ‘self-efficacy’ (e.g. trying again), ‘resilience’ (e.g. celebrating ‘small victories’), and ‘proactivity’ (e.g. with a ‘growth mindset’) ( The Institute for Educational Leadership, 2021b: 9–10). Practices should include: ‘setting the direction’ by building and communicating a vision, identifying ‘shared short-term goals’, ‘creating high expectations’, fostering professional capacity, and maintaining relationships; ‘support desired practice’ such as by ‘building collaborative cultures and distributing leadership’; and fostering shared accountability for outcomes ( The Institute for Educational Leadership, 2014: 5–13). Learning from research and practice, and sharing practice-based lessons, is essential during high uncertainty and major policy change ( Leithwood & McCullough, 2015: 31). Leaders fostering collaboration should engage in ‘courageous conversations’ to challenge ‘current practices’ and seek innovation ( Waterloo Catholic District School Board, 2011: 6).

### What are the barriers or constraints to systems leadership, and how can systems leaders respond?

There are many barriers to systems leadership, relating to: its ambiguity and potential to be co-opted, governance arrangements that are not conducive to collaboration and boundary spanning, and a lack of political support for systems change. There is some debate on how to respond and how influential systems leaders can be, from recommending the humility to recognise a lack of control to seeking the right levers to produce disproportionate change. There is similar debate on the evidence of progress towards more effective systems leadership. There is more agreement on the scale of the task, in relation to limited capacity and progress, albeit with a tendency for positive accounts to relate them to opportunities (while narrating the need for systems leadership). We summarise these barriers in Table 4.

**Table 4.** Barriers to systems leadership.

Ambiguity	Many actors use the language of systems leadership without clarity, energy, or sincerity.
Wicked problems and complex governance	The reasons to foster systems leadership are also the causes of its limited traction or progress.
An unsupportive environment or architecture	Systems leadership has not replaced traditional ideas of authority. High stakes political accountability produces short-termism and blame games. New approaches do not compete well with the old. There are limited opportunities to experiment and learn.
Low capacity and limited training	There is limited tangible support to develop systems leadership capacity across systems.
Unclear or contested responses	There is uncertainty about how to respond to barriers (reflecting debate on the potential influence of leaders in complex systems).
Unclear or contested evidence	There is (and may necessarily be) limited evidence for the success of systems leadership approaches.

***From positive stories to dispiriting experiences of systems leadership***

When focusing on aspiration, we describe stories of the *potential* for systems leadership to produce more effective and democratic governance. For example, if there are meaningful levels of decentralisation and local autonomy, systems leaders come into their own: helping many actors to shift from a focus on delivering top-down aims to working across boundaries to coordinate the design of local policies, and working with stakeholders and citizens expressing a diverse range of views. When focus on policymaking reality, we find that practitioners are navigating contradictory dynamics: they face pressures to deliver on top-down aims *and* perform the role of semi-autonomous leaders ( Bolden *et al.*, 2020: 27).

**Figure 2.**
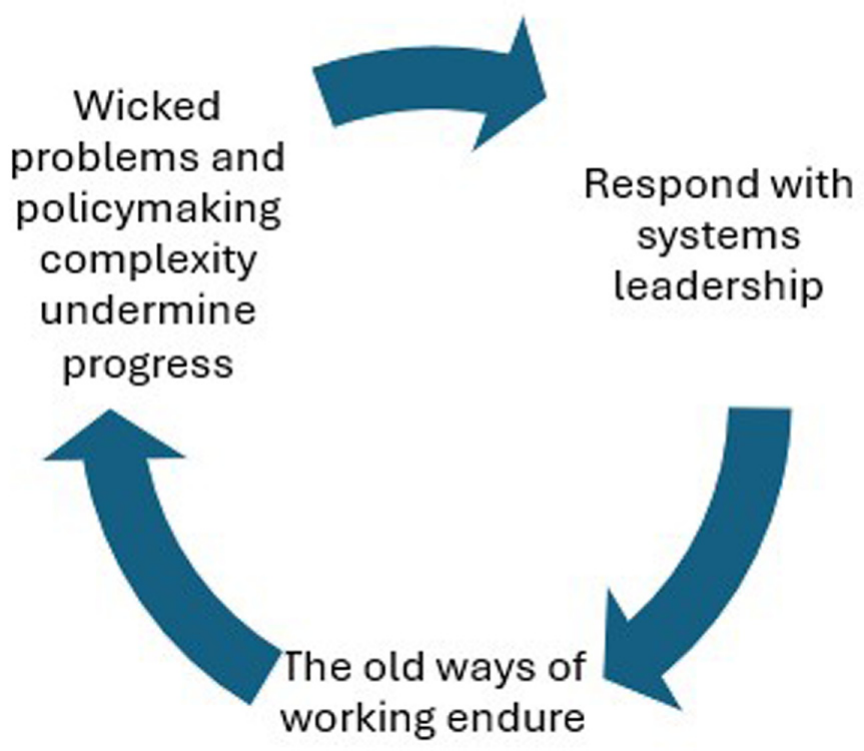
The systems leadership loop.

In other words, ‘systems leadership’ can be used to describe one thing or its opposite. For some, it reflects the pursuit of a positive vision of systems change, driven from the bottom-up to harness the energy of policymakers, practitioners, stakeholders, and citizens to improve health, education, or social services (2020: 29). For others, it reflects the co-option of a yet another new term to maintain old aims: the delivery of top-down policies by systems leaders, or the mild impetus to do things differently without addressing imbalances of power (e.g. national-local, senior-junior, health-other, or practitioner-citizen) (2020: 34). This dynamic can be represented simply in Figure 2 as a continuous cycle of similar problems and responses.

Various accounts present variations on these positive and negative themes. Nguyen *et al.* (2023: 13) find positive narratives from interviews with UK civil servants: a ‘top-down approach to advancing systems thinking adoption’ is essential to its use even if it has not yet taken off ( Nguyen *et al.*, 2023: 13). Further, systems leaders may need to be in senior positions (such as Director General in the UK) or have ‘sufficient standing and authority in Whitehall to be viewed as influential’ ( Aston & Sweetland, 2024: 22; see also Barker, 2016; Education Scotland, 2019: 16; Government Office for Science, 2023). Similarly, Haynes *et al.*’s (2020: 69) interviews with Australian health ministry civil servants found that they enjoyed the opportunity to work closely with ‘great thinkers and practitioners’. In that context, when systems leaders ‘see new opportunities arising just as we see our capacity to exploit them diminishing’ they treat such difficulties as a justification for systems leadership ( Atkinson *et al.*, 2015: 8). Indeed, systems leadership may resonate with ‘an ambitious narrative about public service improvement, including how to create an optimal balance between localism and centralisation’ and a sense that powerful leaders had failed and caused the current crisis ( Ghate *et al.*, 2013a: 9).

More critical accounts describe the use of systems language as a sleight-of-hand by national governments to shift the blame for policy failure onto local policy actors:

“In the last decade, alongside the increased focus on whole systems working, people-centred design, and integration of public services, many countries have faced a significant decline in public funding, whilst demand for services continues to rise … Despite the rhetoric of greater local involvement in decision-making, centralized control mechanisms and decreasing funding greatly limit the capacity of local leaders to deliver services, whilst at the same time shifting accountability and responsibility from the central state to local partners’” ( Bolden *et al.*, 2020: 29).

Mowat (2019: 67–98) expresses the same caution that a national government may use ‘systems leadership’ as a vaguely aspirational aim without reforming top-down structures or providing resources to make meaningful changes. ‘Systems leadership’ becomes a process of co-opting an aspirational term to protect top-down practices.

Such accounts present variations on the theme of low *clarity*, *congruence*, and *capacity* (see Cairney *et al.*, 2022), when vaguely new ways of thinking do not fit with the old ways of doing things, and there are limited resources for change. This problem begins with insufficient clarity to guide preparation: attachment to a vague aspirational term does not allow you to explore the ‘tensions, paradoxes and dilemmas this poses for leaders and their organizations or how they can be addressed’ and may prove to be ‘empty rhetoric’ describing something new to gloss over the old NPM ( Bolden *et al.*, 2020: 26; 34). Consequently, government may be unconducive to ‘systems thinking’ approaches when staff face an ambiguous complexity language, struggle to connect it to the ‘dynamics of policymaking’ and ‘political context’ or ‘policy culture’, especially when faced with factors – including ‘time and resource constraints’ and ‘high turnover’ – that disrupt learning ( Nguyen *et al.*, 2023: 8; see also Barker, 2016: 5–6). People may agree on the aim, but it is hard to: ‘get started’ when there is a lack of clarity on what to do (or a lack of a crisis to focus minds); ‘maintain momentum’ when there is ‘no cookbook’ for collective action and resources for coordination are limited; form meaningful relationships built on the familiarity and trust that comes from long-term engagement; or use trust to encourage ‘vulnerability’ during experimentation ( Bigland *et al.*, 2020: 4–7). Examples of this theme include:

*New systems versus old hierarchies*. In domestic (country level) accounts, progress is ‘painfully slow’ whenever systems leadership is incongruous with routine government business ‘amongst individuals and organizations habituated to working in bureaucracies, where professional expertise, hierarchical position and rules and processes are prevalent’ ( Bolden *et al.*, 2023: 338). Systems leadership may be catching on, but in public services dealing with wicked issues, facing increasing demand but lower resources, and subject to regulations, inspections, and performance measures that undermine collaboration across services for the long-term ( Atkinson *et al.*, 2015: 8). It is also hard to reconcile systems thinking with other forms of leadership based on authority, such as clinical leadership in hospitals ( Onyura *et al.*, 2019: 626). These competing forms of responsibility need to be reconciled, such as to harness ‘clinical expertise and credibility’ but also ‘consultancy functions that share expertise within the wider system; leadership for culture change’ ( Manley *et al.*, 2016: 5; see also Swanwick & McKimm, 2011). Similarly, in public health, systems approaches compete with other approaches to wicked policy problems, such as ‘acute care and epidemiological models that focus on isolating independent actionable causes’, and in policymaking where the uptake of systems leadership is limited ( Haynes *et al.*, 2020: 66 on Australia).

In international accounts, the development of systems leadership in one context may be undermined by another, such as when Rose *et al.* (2024: 14) identify the development of local adaptive capacity among ‘academic biomedical researchers in Africa’ but power imbalances in favour of foreign funders in ‘an environment of research colonialism’. As such, advice for systems leadership relates to the mindsets of participants and the wider environment that can facilitate or constrain new dynamics. Fast and Bennett ( 2020: 12; and Holloway *et al.*, 2020: 23) make a similar point about ‘the dominance of international actors in the leadership and coordination of the humanitarian system’, making ‘local contributions … consistently undervalued’.

*Error versus failure*. An emphasis on curiosity and learning is not a good match to high-stakes policy and high-pressure practice where experiential trial-and-error learning would be equated with failure and blame-based accountability ( Evans *et al.*, 2021: 26).

*A new moral focus lacks capacity or support*. It is difficult to maintain a ‘focused moral purpose’, adapt ‘infrastructures and strategies across time’, and build professional capacity to ‘match shifting demands’ ( Meyers, 2024: 302), especially when a profession of systems leaders has not yet developed ( Dreier *et al.*, 2019: 12). Further, some advocates of systems thinking may exacerbate tensions in government; ‘evangelical researchers’ contribute to ‘painful discussions’ that threaten to ‘alienate policy-makers’, such as by dismissing requests by new audiences for concrete examples ( Haynes *et al.*, 2020: 69–70)

*A burst of ideas and potential but lack of training*. Several studies ( Chavez *et al.*, 2019; DeClerk *et al.*, 2022; Hardin *et al.*, 2022; Thompson & Nelson-Marten, 2011) show that online scenario-based, postgraduate, and bespoke training are *possible*, while Talley and Hull (2023) describe the potential for training on addressing sustainability challenges including ‘climate change, water security, poverty and supply chain resiliency’, and the Centre for Effective Leadership (2023: 1) describes ‘developing the leadership skills and mindsets needed to be effective and adaptable in an increasingly complex government system’. Yet, the need for systems leadership capacity does not correspond to modes of training in field such as nursing, medicine, or public health ( Bigland *et al.*, 2020: 2; Evans *et al.*, 2021: 26; Rose *et al.*, 2024). There are too few people trained in systems leadership; too few people understand what it is or why it is necessary ( O’Malley *et al.*, 2022: 11).

*The impetus for systems leadership also undermines it*. The need for joined-up thinking is undermined by public sector fragmentation and a tendency for weak links between organisations, such as within healthcare ( Manley *et al.*, 2016: 5), across health and local government in England ( Evans *et al.*, 2021: 27), and across UK government departments ( Nguyen *et al.*, 2023: 8).

Such problems have prompted meaningful reflection in leading countries such as New Zealand. For example, the Deputy Prime Minister and Minister for Health (2021: 8) describe a sincere pursuit of ‘system leadership’ for mental health and addiction policy, undermined by a tendency to treat funding as ‘a collection of individual components and not a unified programme of delivery’, and the inability of the Ministry of Health to ‘exercise system leadership over this Programme’ (outside of health policy). The proposed response is to grow and train a workforce with system leadership skills, Ministry ‘stewardship’ over the cross-government programme, and more strategic capacity in ‘planning and reporting’ (2021: 10–11; compare with Hallsworth, 2011: 13 using ‘system stewardship’ to describe a necessary shift of mindset of policymakers). Similarly, the Ministry of Transport (2019: 57) identified a need to ‘Strengthen system leadership, support and coordination’ when addressing road safety, followed by the appointment of a ‘programme director’ to ‘provide cross-system leadership and agency integration’ and monitor performance ( Ministry of Transport, 2022: 82)

***How can systems leaders respond? With humility or heroism?***

Although we have identified a common origin story, it is important not to use it to hide important differences of approach and expectations. Rather, ‘systems thinking’ can inform contrasting ambitions for leaders, from the ability to have a disproportionately strong impact on systems change (finding ‘leverage points’- see Meadows, 1999), to the need for humility to recognise that the requirement of collective action across a system precludes the disproportionate impact of a heroic leader ( Cairney, 2021).

We find examples of each exposition of leadership in complex systems. The strongest ‘leverage point’ assertion is by Reinertsen *et al.* (2008: 6), to describe ‘healthcare organizations and communities’ as complex adaptive systems, and argue that ‘If leaders could choose the right system attributes (“leverage points”) and make small, perhaps difficult, but important changes, very large performance change might result (e.g. to set systems-level performance measures, identify a system-wide change strategy, boost leadership, engage staff and service users, get finance on board, and build capacity for improvement). Similarly, Hagel and Mortensen (2018: 6) argue that ‘the important thing is to achieve the greatest possible impact with the least amount of effort. Successful systems leaders always search for the so-called “leverage points” in the systems they are trying to change – the particular elements that play a disproportionate role in shaping the evolution of the overall system’. In contrast, Bolden *et al.* (2023: 339) cite Stacey’s (2006: 30) warning to avoid a ‘naive form of systems thinking’ in which a leader can step outside of a system to influence it, which “greatly exaggerates the potential for ‘system leaders’ to exert system-wide influence” and underplays the role of emergence from local interactions. Rather, seek ways to contribute effectively to a collective enterprise ( Walker *et al.*, 2022: 3 also warn against the language of levers).

Within this range, we find mildly positive accounts based on documenting personal experiences ( The Leadership Centre, 2015; see also Coffey, 2010). There are also some modest ambitions to facilitate ‘emergence’ as part of a collective enterprise. Marion and Uhl-Bien (2001: 396–406) describe individuals who can *influence* systemic processes in the absence of *control*, producing a language consistent with the former, such as to *foster* or *catalyze* the construction of networks, act as ‘tags’ to symbolise ideas, and ‘drop seeds of emergence’. Further, Lichtenstein and Plowman (2009) suggest that this process begins by understanding four ‘conditions’ for positive outcomes to emerge from complex systems then encouraging ‘leadership behaviours’ that facilitate these processes:

1. ‘Dis-equilibrium state’.
  • There is a notable ‘disruption’ in a system that causes instability, which ‘sparks emergent change processes’.
  • Response: ‘embrace uncertainty’ and encourage system members to reflect on why this disruption has occurred, and ‘surface conflict and create controversy’ to prompt members to ‘generate novel opportunities and solutions’.
2. ‘Amplifying actions’.
  • In this disequilibrium state, one part of a system can amplify action in another. Response: ‘amplify actions’ by fostering ‘experiments and fluctuations’, and ‘encourage rich interactions’ and ‘collective action’ in which many actors amplify ideas or responses by others.
3. ‘Recombination/Self-organization’.
  • Amplifying activity puts pressure on a system to the extent that it ‘can either collapse or re-organize’.
  • Response: foster ‘sensemaking’ and ‘sensegiving’ to encourage the generation of new ‘language and symbols’ that promote ‘correlation’, or a new ‘shared understanding of the system’. Use that new understanding to encourage the ‘recombination’ of systemic resources for a new purpose. Assume the role of ‘tags’, to represent ‘a valued set of behaviors’, draw attention to ‘things that are important’, and symbolize key messages.
4 ‘Stabilizing feedback’.
  • A ‘new emergent order’ is apparent when there is less instability and key parts of a system dampen any impetus for change.
  • Response: balance the pressure for change with the need to ‘integrate local constraints’ that could otherwise foster new forms of instability (2009: 620–1; 622–5).

Lichtenstein and Plowman (2009) recognise the small-N nature of such research and the possibility of cherry-picking experiences. Other small-N studies promote different conclusions, such as when Buchanan *et al.* (2007: 1086) describe a successful project in retrospect without being able to use it to design similar success (beyond developing skills for dealing with ‘fluid’ settings when there appears to be ‘nobody in charge’). Further, larger-N studies in political science emphasise not only the lack of individual ability to influence punctuated change, but also a researcher’s lack of ability to predict these dynamics ( Baumgartner *et al.*, 2023).

***What is the evidence for progress, and how should we interpret the evidence?***

The gap between aspiration and reality is costed into romantic systems narratives, so it is possible to find two contrasting interpretations of current progress. Senge *et al.* (2015: 30) go positive. They note that systems leadership involves ‘Bringing together diverse stakeholders with little history of collaboration, different mental models, and different and even apparently competing aims’, which ‘is a high-risk undertaking’. Then, they relate systems leadership to the ‘discipline’ and commitment of leaders, aided by tools to see the larger system (e.g. systems maps), facilitate reflection and conversation (e.g. ‘peer shadowing’ and ‘learning journeys), and co-create a future vision (e.g. an ‘appreciative inquiry summit’) (2015: 32). Their enduring commitment allows them to develop skills as they ‘learn on the job’, balance their ‘advocacy and inquiry’ roles, reach ‘across boundaries’ to engage, ‘follow the energy’ of the group, learn what tools work for them, reflect with other systems leaders (as we make progress towards ‘critical mass’), and help people avoid ‘fatalism’ as they seek meaningful change in systems (2015: 32–3).

On the other hand, a key tenet of complex systems analysis is to avoid individualising leadership roles. Wicked policy problems and complex policymaking processes are not – and could not be - in the control of even the most tenacious leaders. Further, being uncritically optimistic may lead people to assume that, for example, ‘collaboration is good and that pooling resources will automatically result in positive outcomes’ rather than research what ‘effective collaboration means or looks like’ ( Jang *et al.*, 2016: 88). This problem begins with a tendency to use the phrase ‘systems leadership’ without conceptual clarity or substantive empirical evidence on which to draw ( Begg, 2020: 6; Bigland *et al.*, 2020; Carey *et al.*, 2015; Evans *et al.*, 2021; Fawkes, 2013: 21; Health Foundation, 2010; Miller, 2020: 3; notes that this lack of evidence for effectiveness is a broader feature of leadership training). It can easily become a distracting buzzword, bandied around without clear definition ( Gordon *et al.*, 2023). This problem with clarity may seem academic, but could explain a shaky foundation for concerted action and the potential for demoralisation.

**Example: systems leadership for health and social care in England**

In that context, the much-studied process for health and social care integration (and public service reform) in England is a key reference point and source of contested ideas of progress. The most positive account of progress is by NHS England (2018: 58) when reflecting on the use of systems leadership for its major change programme in the National Health Service:

‘This way of working has a solid evidence base, encompassing leadership thinking, research and practice from around the world’ … the ‘national systems leadership programme … backed by the NHS, local and national government, social care, public health and other sectors … has helped people make real progress in changing how they think, how they behave and what happens on the ground as a result, as evidenced in independent evaluation and in reports from the places themselves’ (here, ‘evaluation’ refers to Ghate *et al.* (2013a) and The Leadership Centre (2015)).

Other positive accounts describe the benefits of systems leadership without defining it or explaining the mechanisms of change, such as: a ‘systematic review of complex healthcare interventions … emphasised the quality of systems leadership as an important aspect in the success of interventions’ ( Baxter *et al.*, 2019: 6); and, ‘The investment in a clinical leadership programme focused on systems leadership for quality generates value for the NHS’ ( Malby *et al.*, 2018: 793). Similarly, De Brún *et al.*’s (2019: 15–17) systematic review of ‘interventions to develop collectivistic leadership in healthcare settings’ (not systems leadership in particular) identified a range of approaches reporting positive outcomes (e.g. a higher sense of empowerment and lower staff turnover) – including ‘co-design’, ‘co-leadership’, ‘service improvement’, ‘team training’, and ‘individual team development’ interventions – but a ‘paucity of research’ of sufficient transparency and quality.

Other accounts of health and social care systems change in England suggest that it exemplifies the gulf between the frequent promotion of systems leadership ( Local Government Association, 2018; Miller, 2020: 3) and the lack of conduciveness for this approach in the system. There is an iterative discussion of barriers and opportunities, in which reports describe the value of systems thinking and leadership, acknowledge the barriers and lack of progress, then reflect on how to use systems leadership principles or practices to respond (see Figure 2).

For example, Timmins’ (2015) summary of interviews with systems leaders describes it as: ‘not easy’, time consuming, and not supported well enough in terms of organisational and staff learning, financial allocations, or current incentives in healthcare organisations:

‘The pressures of regulation, financial balance and organisational targets are still leading people and organisations to draw in their horns and ‘hunker down’ to survive, rather than seeing the way forward in terms of changes that will alter and, in some cases, downsize what their organisation does. Regulation, in particular, needs to be reformed. All too often, the current system gets in the way of system change, and thus system leadership’.

Gordon *et al.*’s (2023) interviews with healthcare system chief executives suggest that their systems do not support systems leadership, since elements such as financial allocations, regulations, and organisational structures are geared towards hierarchy, performance management, and holding senior leaders responsible. Leaders face contradictory dynamics that encourage systems leadership rhetorically but ‘sacks’ chief executives for failure to meet short-term targets ( Gordon *et al.*, 2023, citing Anandaciva *et al.*, 2018: 31–2). The legacy of high stakes internal market competition is two-fold: competition accentuated the ‘heroic’ manager image so prominent in politics and clinical work ( Fillingham & Weir, 2014: 14); and, it pitted managers against each other ( Anandaciva *et al.*, 2018: 57; 64). Consequently, many people in leadership positions ‘don’t have a collaborative bone in their body’, much of the language of recruitment in key positions is ‘about looking for someone who still runs a tight ship’, and many are willing to engage in training and learning but subject to ‘operational pressures’ that rules it out ( Anandaciva *et al.*, 2018: 57; 64).

Similar tensions are apparent between and within health bodies and local government ( The Leadership Centre, 2015: 5; Miller, 2020: 3) or across the public service. Walker *et al.*’s (2022: 3) interviews with local government officers highlights barriers to ‘the adoption of system thinking’ including low capacity and funding, a ‘culture of risk aversion among many in leadership positions’ and ‘institutional disconnect between agencies involved in health and wellbeing’. Taylor *et al.* (2020: 56) describe obstacles to new ways of thinking and working across social movements:

‘initial challenges were received from organizations that did not understand the approach and the structure of an informal partnership to drive change. Traditionally, public-sector bodies and even many third-sector organizations in the UK operate with a focus on hierarchical decision-making and governance structures, which are counter to an approach founded upon systems leadership. Systems leadership is an “art” approach to change making and not a “science.” It is founded in ways of feelings, perceiving, thinking, relating, doing and being, where strong relationships and trust are key’.

For example, they suggest that some senior public sector leaders challenged this approach to their social movement perhaps because they feared the loss of their own power (2020: 56) or knew that they would still be subject to harsher accountability regardless of their additional role.

Other accounts from practitioners invested in systems leadership (as facilitators or trainers) express some disappointment with progress, including:

‘What is shocking is how little time is being put aside for reflection in many major systems changes and how powerful the ‘wilful blindness’ has already become. I have been working in a number of places where those people exposed to system leadership thinking, and therefore reflecting hard, are struggling to get other senior leaders around them to reflect or learn’ ( Goss, 2021: 15 on UK policymaking).

Similarly, The Leadership Centre (2015: 5) describes ‘scepticism among senior leaders’: they see systems leadership as another fad and have witnessed demoralising ‘historical, failed attempts to re-engineer the system’.

## Discussion: actionable lessons from academic and practitioner research

The term ‘systems leadership’ has traction in leadership studies and has become a buzzword in some fields ( Bigland *et al.*, 2020: 2; Smith *et al.*, 2021). It shares with ‘systems thinking’ the sense that scholars ‘extol the benefits of introducing systems science’ but often ‘contain very little detail about what this would entail’ ( Carey *et al.*, 2015: 3). This initial ambiguity can be helpful to generate widespread support for a common endeavour, but with high potential for agreement to fizzle out when actors seek more clarity ( Cairney *et al.*, 2022).

In that context, we seek to synthesise insights from the literature to find clarity on systems leadership. Our first review described a paucity of advice and the need to synthesise insights to piece together a coherent story of collaborative policymaking. This second review has almost the opposite problem: there is so much disparate advice (see Results) that it is hard to piece together into one coherent story. Nevertheless, we can produce (in Table 5) actionable lessons that are comparable to the ‘five features of collaborative policymaking’ ( Cairney & Toomey’s, 2024a).

**Table 5.** Five features of systems leadership.

A systems leadership mindset	Reject heroic top-down leadership Collaborate to fulfil a vision and moral purpose Focus on the big picture Be flexible, curious, and open to learning
A sophisticated understanding of wicked problems and policymaking complexity	Learn how wicked problems transcend traditional silos, and how to collaborate across boundaries Anticipate the dynamics of complex policymaking systems
Systems leadership capacity	Clarify key principles, roles, and competencies Foster attributes such as humility and compassion Foster skills such as enabling, brokerage, facilitation, evaluation, conflict resolution, and political astuteness
A supportive architecture	Maintain support for systems leadership cultures in politics and performance management Secure the time and space for training and development Protect a common vision or focus, such as Place or Equity.
Innovation and learning	Engage in continuous trial-and-error learning Build and share an evidence base for success

### A systems leadership mindset

This new mindset begins by rejecting the old focus on heroic leaders and top-down approaches in favour of more collaborative leadership. Collaboration may begin by coming together to establish a common vision and moral purpose, such as to facilitate more effective and equitable systems, then working together to share responsibility for its delivery. Throughout, the systems mindset involves focusing on the big picture of systemic dynamics and looking for opportunities to facilitate systems change (while being realistic about the impact of individual action). Given the value of collaboration, and the need to respond to profound uncertainty, the aim is not to assign responsibility for a small group of exceptional leaders, able to unlock systems to leverage disproportionate change. Rather, the mindset is of facilitating conversation, being curious and open-minded enough to consider multiple perspectives, and to learn from others, aided by systems leadership tools.

### A sophisticated understanding of wicked problems and policymaking complexity

A systems mindset is essential to address complex policy problems and processes. Most of the world’s most urgent and important policy problems are ‘wicked’, or not amenable to technocratic policy analysis and simple solutions by one centre of government. Rather, many policy actors – across governments and inside/outside of government – need to collaborate to generate a common understanding of problems and how to respond. This action takes place in complex systems that defy our full understanding far less control. Systems dynamics include: the same actions or ideas can be magnified or dampened; the appearance of regular patterns of behaviour belies the potential for major disruptions; and, outcomes emerging in the absence of central control. A systems mindset includes the need to anticipate such dynamics, get comfortable with uncertainty and ambiguity, and seek windows of opportunity to support systems change.

### Systems leadership capacity

This new approach needs to be adopted by many people across systems, necessitating a profound boost to capacity. It requires the identification and communication of key principles of systems leadership, and essential roles and competencies, and to value relational attributes such as humility, self-reflection and awareness, empathy, and compassion. It requires skills training to: meaningfully delegate and enable many others (while making sure that everyone has a clear sense of purpose), facilitate collaborative discussion and action, generate new ways to evaluate and account for collective action, balance creative tension and conflict, and work effectively in political systems to navigate the political beliefs and tensions that may undermine collective action.

### A supportive architecture

This new approach needs continuous and substantive political and organisational support to generate a systems leadership culture: giving people the time and space to train, develop, and learn; using accountability and performance management measures that encourage people to innovate and take risks then learn from trial-and-error (rather than blaming individuals for failure); and generating then maintaining a common vision or focus (such as to foster equitable policy processes and outcomes and/or focus on ‘place’) to concentrate collaborative efforts and identify agreed practices and aspirations.

### Innovation and learning

For some, the value of systems leadership is self-evident or an attractive leap of faith, backed by learning from personal experience and shared stories of facing or overcoming challenges. For others, the ideas seem vague and aspirational without being backed by ‘hard’ evidence of its real-world value. For all, the role of generating and sharing evidence is essential, to help its advocates learn from good practices, and demonstrate to other audiences that the shift in approach will produce tangible benefits.

### Are these features merely aspirational or achievable?

It is straightforward to produce a coherent account of aspirational systems leadership but not to demonstrate its progress or achievability. We find many stories of a shift in thinking among a growing group of scholars and practitioners, but without demonstrating that it has taken off in the governments or networks where these ideas would be used. There is more written on what systems leaders should do than how to do it ( Begg, 2020: 16–17). We find lists of what is required of policy actors facing wicked problems and policymaking complexity, accompanied by hopes for the greater effectiveness and equity of this approach, but limited evidence of the payoffs. The vague aspirational language of systems leadership exacerbates the problem: how can we demonstrate the benefits of this approach when we are not sure what it is, or how to do it? The problem grows when we see what is required to support systems leadership. The literature adds up to a shopping list of attributes, skills, strategies, principles, competences, or roles required to produce sufficient systems leadership capacity. This literature has produced the potential to create the world’s longest leadership training course, bolstered by the maxim that any barrier to systems leadership is an opportunity to train and learn. Finally, the problem peaks when we read reflective accounts of limited progress. Some reflect that the reasons to adopt systems leadership also explain its barriers: power hoarding by central governments, the fragmentation of government or governance, and competition between organisations (or siloed action) contribute to a policymaking environment that is not conducive to new forms of collaboration or rewarding of new ways of thinking and acting ( Figure 2).

One frequent – and reasonable – response is to flip this narrative to describe systemic barriers as a justification for systems leadership. Further, there is also considerable evidence of progress to justify some investment in a new approach. However, these case studies of success come largely in the form of external testimonies about exceptional individuals (e.g. Senge *et al.*, 2015 on Nelson Mandela) or the personal testimonies of systems leaders who have bought into the process (e.g. Wenger-Trayner & Wenger-Trayner, 2021). Further, in very few real-world accounts can we find a clear connection between the authors’ conception of complex systems and the effectiveness of a systems leadership response (one exception is by Lichtenstein and Plowman (2009) who recognise the limitations of their small-n approach). Rather, at that stage of reflection on progress, the idea of a system becomes vague and metaphorical and the focus returns to the competencies and actions of policy actors. Whether or not we need the initial narrative of systems thinking – translating insights from complexity theory – to help inform or explain this action remains an open question (see also Jackson, 2024: 32 on the incoherence of complexity theory when it connects to so many ideas). Overall, we find an approach to leadership with limited clarity that is not congruent with governance routines and lacks professional, organisational, and systemic capacity (which might be described by systems leadership advocates as a challenge worth facing).

## Conclusion

This paper presents our second review to synthesise academic and practitioner insights to offer pragmatic advice to policymakers seeking more effective collaborative policymaking. We relate this advice to a story of policy and policymaking complexity in which policy problems *require* meaningful collaboration by actors spread across multiple organisations and policy sectors but may struggle to secure meaningful collective action. The first review found two kinds of aspirational advice, relating collaboration to ‘rationalist policy processes and the protection of democratic ideals’ ( Cairney & Toomey, 2024a: 22). Here, we found a rejection of rationalist or technocratic ambitions as part of the systems leadership origin story. This story combines elements of many new approaches to leadership with a focus on complex systems. Here, the main aspirations are for a more inclusive and democratic collaboration in which leadership is spread across organisations and policymaking systems. It is sometimes accompanied by a sense of realism about what can be done in relation to wicked problems in policy processes that are not conducive to power distribution and collaboration.

We summarise our interpretation of useful advice as follows:

1. *Foster a systems leadership mindset*.
  •   Reject heroic top-down leadership.
  •   Embrace collaboration to produce and deliver a collective vision.
  •   Focus on the big picture and be comfortable with systemic uncertainty and ambiguity.
  •   Facilitate dialogue, consider multiple perspectives, and learn from others.
2. *Interrogate wicked problems and navigate policymaking complexity*.
  •   Collaborate to generate a collective understanding of wicked problems
  •   Share responsibility for a concerted response.
  •   Anticipate the dynamics of complex policymaking systems
  •   Seek opportunities to work with many others to facilitate systems change.
3. *Boost systems leadership capacity*.
  •   A distributed and collaborative approach requires a large and well-trained profession.
  •   Systems leaders are able to communicate key values and principles, fulfil essential roles and competencies, and practice and reward relational approaches.
  •   Essential skills include meaningful delegation and enabling, facilitation, to foster creative tension and conflict, and to work effectively in competitive political systems.
4. *Maintain a supportive architecture*.
  •   Systems leadership does not come naturally. It needs high and sustained political support.
  •   Maintain a culture of collaboration, development, and reflection.
  •   Reward innovation.
  •   Maintain collective support for a common vision.
5. *Embed innovation and learning to provide evidence of progress*.
  •   Shared stories of facing barriers and having some success are essential to the morale and understanding of actors performing systems leadership.
  •   Substantive evidence is essential to more sceptical audiences and the policymakers required to provide political, financial, and organisational support for systems leadership approaches.

There is no detailed how-to guide, since advocates stress the need to understand the complex context in which they engage and adapt accordingly. Rather, think of systems leadership scholarship and practice as an ongoing dialogue in which there is often productive debate about how to conceptualise and respond to complexity. First, when reflecting on complexity theory, some: emphasise the need for humility in the absence of certainty or control; or seek to identify levers for disproportionate change and inspire others with stories of success. Second, when reflecting on progress so far, some tell: dispirited stories of low clarity on what systems leadership is, low congruence with business-as-usual governance, and low capacity to make a difference in fragmented policymaking systems; or, inspirational tales of individual success and opportunities for systems change. In other words, for its advocates, the limited translation of systems leadership ideas into real-world practice is a reason to redouble efforts to turn barriers into opportunities and potential into reality.

## Ethics and consent

Ethical approval and consent were not required.

## Data Availability

No data associated with this article. Open Science Framework: Systems leadership This project contains the following extended data: - Structured bibliography Qualitative Systematic Review https://osf.io/4gz7b, https://doi.org/10.17605/OSF.IO/HR769 ( Cairney & Toomey, 2024b) - Study Protocol https://osf.io/z4kgr, https://doi.org/10.17605/OSF.IO/HR769 ( Cairney & Toomey, 2024b) Data are available under the terms of the Creative Commons Attribution 4.0 International license (CC-BY 4.0) Open Science Framework: Systems leadership The project contains the following additional information: PRISMA checklist https://osf.io/6szqk, https://doi.org/10.17605/OSF.IO/HR769 ( Cairney & Toomey, 2024b)

## References

[ref-1] Acosta JD Madrigano J Chandra A : Adapting to adversity amid a global pandemic: stakeholder insights about progress and next steps for taking integrative action to build resilient systems. (Washington DC: RAND Corporation),2021. Reference Source

[ref-2] AIS: AIS leadership and culture: stakeholder management. (Canberra: Australian Institute of Sport),2021. Reference Source

[ref-3] Anandaciva S Ward D Randhawa M : Leadership in the NHS: delivering the impossible. (London: King's Fund),2018. Reference Source

[ref-4] Ansell C Gash C : Stewards, mediators, and catalysts: toward a model of collaborative leadership. * Innov J.* 2012;17(1):1–21. Reference Source

[ref-270] Aoki N Tay M Rawat S : Whole-of-government and joined-up government: a systematic literature review. *Public Administration.* 2024;102(2):733–752. 10.1111/padm.12949

[ref-5] Arnold RD Wade JP : A complete set of systems thinking skills. *INSIGHT.* 2017;20(3):9–17. 10.1002/inst.12159

[ref-6] Aston J Sweetland J : From sidelined to systemic: the role of whitehall’s chief scientific advisers. (London: Reform),2024. Reference Source

[ref-7] Atkinson J Loftus E Jarvis J : The art of change making. (London: Leadership Centre),2015. Reference Source

[ref-8] Auld G Bernstein S Cashore B : Managing pandemics as super wicked problems: lessons from, and for COVID-19 and the climate crisis. *Policy Sci.* 2021;54(4):707–728. 10.1007/s11077-021-09442-2 34803187 PMC8596365

[ref-9] Avolio BJ Walumba FO Weber TJ : Leadership: current theories, research, and future directions. *Annu Rev Psychol.* 2009;60:421–49. 10.1146/annurev.psych.60.110707.163621 18651820

[ref-10] Barkell NP Snyder SS : Just culture in healthcare: an integrative review. *Nurs Forum.* 2021;56(1):103–111. 10.1111/nuf.12525 33231884

[ref-11] Barker P : Building policy capability in the ministry for culture and heritage. (Wellington: Ministry for Culture and Heritage and The Policy Project),2016. Reference Source

[ref-12] Batliwala S Friedman M : Achieving transformative feminist leadership a toolkit for organisations and movements. (New Delhi: CREA),2014. Reference Source

[ref-13] Baumgartner FR Jones BD Mortensen PB : Punctuated equilibrium theory: explaining stability and change in public policymaking. In: Weible, C. (ed) *Theories of the policy process.* 5th ed. (London: Routledge),2023;65–99. 10.4324/9781003308201-4

[ref-14] Baxter S Johnson M Chambers D : Towards greater understanding of implementation during systematic reviews of complex healthcare interventions: the Framework for Implementation Transferability Applicability Reporting (FITAR). *BMC Med Res Methodol.* 2019;19(1):80. 10.1186/s12874-019-0723-y 30999848 PMC6472061

[ref-15] Begg H : Systems leadership rapid review. (London: UK Cabinet Office National Leadership Centre),2020. Reference Source

[ref-16] Beharrell W Richards L Driscoll M : What can we learn about systems leadership from the building of a Welsh surge hospital and how might this be applied beyond the current COVID-19 response? *BMJ Lead.* 2021;5(2):93–97. 10.1136/leader-2020-000311 37579292

[ref-17] Benington J Hartley J : “Whole systems go!” improving leadership across the whole public service system. (London: National School of Government),2009. Reference Source

[ref-18] Bertini C : Leading change in United Nations Organizations. (Chicago: The Chicago Council on Global Affairs),2019. Reference Source

[ref-19] Bigland C Evans D Bolden R : Systems leadership in practice: thematic insights from three public health case studies. *BMC Public Health.* 2020;20(1): 1735. 10.1186/s12889-020-09641-1 33203397 PMC7673088

[ref-20] Blankart CR De Gani SM Crimlisk H : Health literacy, governance and systems leadership contribute to the implementation of the one health approach: a virtuous circle. *Health Policy.* 2024;143: 105042. 10.1016/j.healthpol.2024.105042 38518391

[ref-21] Bolden R : Distributed leadership in organizations: a review of theory and research. *Int J Manag Rev.* 2011;13(3):251–269. 10.1111/j.1468-2370.2011.00306.x

[ref-22] Bolden R : Systems leadership: Pitfalls and possibilities.(London: National Leadership Centre),2020. Reference Source

[ref-25] Bolden R Gulati A Edwards G : Mobilizing change in public services: insights from a systems leadership development intervention. *Int J Public Admin.* 2020;43(1):26–36. 10.1080/01900692.2019.1604748

[ref-26] Bolden R Kars-Unluoglu S Jarvis C : Paradoxes of multi-level leadership: insights from an integrated care system. *J Change Manag.* 2023;23(4):337–357. 10.1080/14697017.2023.2234388

[ref-24] Bolden R O’Regan N : Leadership and creativity in public services: an interview with Lord Michael Bichard, chair of the national audit office. *J Manag Inquiry.* 2018;27(1):45–51. 10.1177/1056492616688088

[ref-27] Bryson J Sancino A Benington J : Towards a multi-actor theory of public value co-creation. *Public Manag Rev.* 2017;19(5):640–654. 10.1080/14719037.2016.1192164

[ref-28] Buchanan DA Addicott R Fitzgerald L : Nobody in charge: distributed change agency in healthcare. *Hum Relat.* 2007;60(7):1065–1090. 10.1177/0018726707081158

[ref-29] Cady P Heykoop C : Strengthening health system leadership in practice. *Healthc Manage Forum.* 2024;37(3):128–132. 10.1177/08404704231209945 37977152 PMC11044504

[ref-30] Cairney P : Complexity theory in political science and public policy. *Polit Stud Rev.* 2012;10(3):346–358. 10.1111/j.1478-9302.2012.00270.x

[ref-31] Cairney P : Policy concepts in 1000 words: complex systems. *Paul Cairney: Politics & Public Policy*.2013; (A: 11.11.24). Reference Source

[ref-32] Cairney P : The politics of policy analysis.(London: Palgrave Pivot),2021. 10.1007/978-3-030-66122-9

[ref-33] Cairney P : An academic story of contemporary policy and policymaking problems. *Paul Cairney: Politics & Public Policy*.2024a; (A: 14.8.24). Reference Source

[ref-34] Cairney P : Policy analysis in 750 words: three systems evaluation. *Paul Cairney: Politics & Public Policy*.2024b; (A: 13.11.24). Reference Source

[ref-35] Cairney P Geyer R : Introduction. In: (eds) Geyer, R. and Cairney, P. *Handbook on Complexity and Public Policy*. (Cheltenham: Edward Elgar),2015;1–16. 10.4337/9781782549529.00006

[ref-36] Cairney P Kippin S : The future of education equity policy in a COVID-19 world: a qualitative systematic review of lessons from education policymaking [version 2; peer review: 2 approved]. *Open Res Eur.* 2022;1:78. 10.12688/openreseurope.13834.2 37645089 PMC10445953

[ref-37] Cairney P Kippin S : Politics and policymaking in the UK. (Bristol: Bristol University Press),2024. 10.56687/9781529222371

[ref-38] Cairney P Toomey C : Collaborative policymaking: a qualitative systematic review of advice for policymakers [version 1; peer review: 3 approved]. *Open Res Eur.* 2024a;4:204. 10.12688/openreseurope.18440.1 39553481 PMC11568374

[ref-39] Cairney P Toomey C : OSF Systems Leadership. *Open Sci Framework.* 2024b. 10.17605/OSF.IO/HR769

[ref-40] Cairney P Heikkila T Wood M : Making Policy in a Complex World.(Cambridge: Cambridge University Press),2019. 10.1017/9781108679053

[ref-41] Cairney P St Denny E Boswell J : Why is health improvement so difficult to secure? [version 2; peer review: 2 approved]. *Open Res Eur.* 2022;2:76. 10.12688/openreseurope.14841.2 37645286 PMC10445925

[ref-42] Cairney P St Denny E Mitchell H : The future of public health policymaking after COVID-19: a qualitative systematic review of lessons from Health in All Policies’ [version 2; peer review: 2 approved]. *Open Res Eur.* 2021;1:23. 10.12688/openreseurope.13178.2 37645203 PMC10445916

[ref-43] Cairney P Timonina I Stephan H : How can policy and policymaking foster climate justice? A qualitative systematic review [version 2; peer review: 2 approved]. *Open Res Eur.* 2023;3:51. 10.12688/openreseurope.15719.2 38106639 PMC10724653

[ref-44] Carey G Malbon E Carey N : Systems science and systems thinking for public health: a systematic review of the field. *BMJ Open.* 2015;5(12): e009002. 10.1136/bmjopen-2015-009002 26719314 PMC4710830

[ref-45] Caro DH : Towards transformational leadership: the nexus of emergency management systems in Canada. *Int J Emerg Manag.* 2016;12(2):113–135. 10.1504/IJEM.2016.076631

[ref-46] Castelyn C : Leadership in healthcare during a pandemic: for a systems leadership approach. *Front Public Health.* 2024;12: 1361046. 10.3389/fpubh.2024.1361046 38841655 PMC11150619

[ref-47] Centre for Effective Leadership: Public policy leadership forum. (Ottowa: University of Ottowa),2023. Reference Source

[ref-48] Chavez F Kelly T Kunisch JR : Systems leadership doctor of nursing practice: global relevance. *Int Nurs Rev.* 2019;66(4):482–489. 10.1111/inr.12527 31206651

[ref-49] Chow AS : One educational technology colleague’s journey from dotcom leadership to university e-learning systems leadership: merging design principles, systemic change and leadership thinking. *TechTrends.* 2013;57(5):64–73. 10.1007/s11528-013-0693-6

[ref-50] Clarke JM Waring J Bishop S : The contribution of political skill to the implementation of health services change: a systematic review and narrative synthesis. *BMC Health Serv Res.* 2021;21(1): 260. 10.1186/s12913-021-06272-z 33743695 PMC7981881

[ref-51] Club de Madrid and Edelman (Working Group 3 of the Global Commission on Democracy and Emergencies): Effective leadership and democratic culture. Building trust between citizens and public institutions in emergency situations. (Madrid: Club de Madrid),2021. Reference Source

[ref-52] Coffey G : A systems approach to leadership: how to create sustained high performance in a complex and uncertain environment. (Berlin: Springer),2010. 10.1007/978-3-642-01194-8

[ref-53] Crosby B : Theoretical foundations of integrative leadership. *Integral Leadership Review.* 2008;1–6. Reference Source

[ref-54] De Brún A O’Donovan R McAuliffe E : Interventions to develop collectivistic leadership in healthcare settings: a systematic review. * BMC Health Serv Res.* 2019;19(1): 72. 10.1186/s12913-019-3883-x 30683089 PMC6347820

[ref-55] DeClerk LC LaBorde PJ Hughes MF : An online COVID-19 simulation to explore interprofessional collaboration and foster systems leadership competencies in doctor of nursing practice students. *Nurs Educ Perspect.* 2022;43(3):193–195. 10.1097/01.NEP.0000000000000909 35482403

[ref-56] Department of the Prime Minister and Cabinet: Strategic intentions 2017–21. (Wellington: New Zealand Government),2017. Reference Source

[ref-57] Deputy Prime Minister and Minister for Health: Mid-term review of the 2019 mental health package. (Wellington: New Zealand Government),2021. Reference Source

[ref-58] Dickson G : Transformations in Canadian health systems leadership: an analytical perspective. *Leadersh Health Serv.* 2009;22(4):292–305. 10.1108/17511870910996132

[ref-59] Dolezal C : Collaborative leadership: forging a system in pierce county. (Minnesota: Optum),2014. Reference Source

[ref-60] Drath WH McCauley CD Palus CJ : Direction, alignment, commitment: toward a more integrative ontology of leadership. *Leadersh Q.* 2008;19(6):635–653. 10.1016/j.leaqua.2008.09.003

[ref-61] Dreier L Nabarro D Nelson J : Systems leadership for sustainable development: strategies for achieving systemic change.(Harvard Kennedy School: CR Initiative),2019. Reference Source

[ref-62] Edmonstone JD : Beyond healthcare leadership? The imperative for health and social care systems. *Leadersh Health Serv (Bradf Engl).* 2020;33(4):351–363. 10.1108/LHS-02-2020-0005 33635022

[ref-63] Education Scotland: Closing the poverty related attainment gap during a period of significant change.(Edinburgh: Education Scotland),2019; (A: 5.11.24). Reference Source

[ref-64] Elliott I Sinclair C Hesselgreaves H : Leadership of integrated health and social care services. *Scott Aff.* 2020;29(2):198–222. 10.3366/scot.2020.0316

[ref-65] Emmenegger P Graf L Trampusch C : The governance of decentralised cooperation in collective training systems: a review and conceptualisation. *Journal of Vocational Education and Training.* 2019;71(1):21–45. 10.1080/13636820.2018.1498906

[ref-66] Enderton AE Bregendahl CM Topaloff AS : Shepherding community engagement to strengthen the local food system in Northeast Iowa. *J Agric Food Syst Community Dev.* 2017;7(2):85–100. 10.5304/jafscd.2017.072.011

[ref-67] Evans D Bolden R Jarvis C : How do you develop systems leadership in public health? Insights from a scoping study. *Public Health.* 2021;196:24–28. 10.1016/j.puhe.2021.04.033 34134012

[ref-68] Fast L Bennett C : From the ground up. It's about time for local humanitarian action.(London: ODI Global),2020. Reference Source

[ref-69] Fawkes S : Leadership for systems change in preventive health – Review of the literature and current activity.(Victoria, Australia: Victorian Department of Health),2013; (A: 27.10.24). Reference Source

[ref-70] Fillingham D Weir B : System leadership: lessons and learning from AQuA's integrated care discovery communities.(London: The King’s Fund),2014. Reference Source

[ref-71] Gale N Dowswell G Greenfield S : Street-level diplomacy? Communicative and adaptive work at the front line of implementing public health policies in primary care. *Soc Sci Med.* 2017;177:9–18. 10.1016/j.socscimed.2017.01.046 28152422

[ref-72] Geyer R : Can complexity move UK policy beyond “Evidence-Based policy making” and the “Audit Culture”? Applying a “Complexity Cascade” to Education and health policy. *Polit Stud.* 2012;60(1):20–43. 10.1111/j.1467-9248.2011.00903.x

[ref-74] Ghate D : From programs to systems: deploying implementation science and practice for sustained real world effectiveness in services for children and families. *J Clin Child Adolesc Psychol.* 2016;45(6):812–826. 10.1080/15374416.2015.1077449 26566999

[ref-73] Ghate D Lewis J Welbourn D : Systems leadership: exceptional leadership for exceptional times: synthesis paper (Executive Summary). (Nottingham: Virtual Staff College),2013a; (A: 27.10.24). Reference Source

[ref-273] Ghate D Lewis J Welbourn D : Systems leadership: exceptional leadership for exceptional times: synthesis paper (Full report). (Nottingham: Virtual Staff College),2013b; (A: 27.10.24). Reference Source

[ref-75] Goldstein J Hazy J Lichtenstein B : Complexity and the nexus of leadership.(London: Palgrave),2010. Reference Source

[ref-76] Gordon B Thomas MG Darzi A : Systems leadership: how chief executives manage tension between organisation and system pressures. *BMJ Lead.* 2023;7(1):72–4. 10.1136/leader-2021-000564 37013886

[ref-77] Goss S : Systems leadership: a view from the bridge.(London: OPM),2021. Reference Source

[ref-78] Government Office for Science: Systems leadership guide: how to be a systems leader.12 Jan,2023; (A: 1.11.24). Reference Source

[ref-79] Greenleaf RK : The servant as leader.(Indianapolis: Greenleaf Centre for Servant Leadership),1970. Reference Source

[ref-80] Grey P Bettiol S Quinn W : Applying systems leadership and participatory action research in developing a water contamination management tool. *Aust J Rural Health.* 2023;31(1):70–79. 10.1111/ajr.12912 35920601

[ref-81] Grint K : Clumsy solutions for wicked problems: decision-making in uncertainty and the role of systems leadership. (London: National Leadership Centre),2020. Reference Source

[ref-82] Gulati K Busari J : Vaccinating a billion people against COVID-19: India’s quest for systems leadership in exceptional times. *Leadersh Health Serv (Bradf Engl).* 2022;35(1):137–148. 10.1108/LHS-05-2021-0045 34787981

[ref-83] Gupta SS Sherif V Zhu X : Re-examining state part C early intervention program coordinators' practices through a positive lens on leadership: a qualitative secondary analysis. *Qual Rep.* 2023;28(2):517–543. 10.46743/2160-3715/2023.4786

[ref-84] Hagel J Mortensen G : Systems leadership and platforms: how to mobilize people to transform systems and build the platforms to scale these efforts. (Geneva: World Economic Forum), 2018. Reference Source

[ref-85] Hallsworth M : System stewardship. (London: Institute for Government), 2011. Reference Source

[ref-86] Hardin LM Gurley LE Cortes C : Innovative team approach for achieving dnp program competencies for distance learners during the COVID-19 pandemic. *Nurs Educ Perspect.* 2022;43(5):318–320. 10.1097/01.NEP.0000000000001007 35791052

[ref-87] Hartley J Parker S Beashel J : Leading and recognizing public value. *Public Admin.* 2019;97(2):264–278. 10.1111/padm.12563

[ref-88] Haynes A Garvey K Davidson S : What can policy-makers get out of systems thinking? Policy partners’ experiences of a systems-focused research collaboration in preventive health. *Int J Health Policy Manag.* 2020;9(2):65–76. 10.15171/ijhpm.2019.86 32124590 PMC7054651

[ref-89] Hazy J Goldstein J Lichtenstein B : Complex systems leadership theory: an introduction.In: Hazy, J., Goldstein, J., and Lichtenstein, B. (eds): *Complex systems leadership theory. *(Boston, MA: ISCE),2007. Reference Source

[ref-90] Health Foundation: Evidence scan: complex adaptive systems. (London: Health Foundation),2010. Reference Source

[ref-91] Hjern B Porter DO : Implementation structures: a new unit of administrative analysis. *Organ Stud.* 1981;2(3):211–27. 10.1177/017084068100200301

[ref-92] Hobbs C Midgely G : How systems thinking enhances systems leadership. (London: National Leadership Centre),2020. Reference Source

[ref-93] Holloway K Barbelet V Meral AG : Collective approaches to communication and community engagement. (London: ODI Global),2020. Reference Source

[ref-94] Hulks S Walsh N Powell M : Leading across the health and care system: lessons from experience. (London: King's Fund),2017. Reference Source

[ref-95] Hunter DJ : Leading for health and wellbeing: the need for a new paradigm. *J Public Health (Oxf).* 2009;31(2):202–4. 10.1093/pubmed/fdp036 19372148

[ref-96] Inland Revenue: Improving policy capability. (Wellington: Inland Revenue and The Policy Project),2021. Reference Source

[ref-97] Jackson MC : Critical systems thinking: a practitioner’s guide. London: Wiley,2024. 10.1002/9781394203604

[ref-98] Jang HS Valero JN Jung K : Effective leadership in network collaboration: lessons learned from continuum of care homeless programs. (Washington DC: IBM Centre for the business of government),2016. Reference Source

[ref-99] Jones L Fulop N : The role of professional elites in healthcare governance: exploring the work of the medical director. *Soc Sci Med.* 2021;277: 113882. 10.1016/j.socscimed.2021.113882 33848720 PMC8135118

[ref-100] Koopmans E Provencher L Irving L : Weaving a new blanket together: lessons on compassionate leadership and engagement from a virtual regional summit on early childhood wellness in northern communities of British Columbia, Canada. *Res Involv Engagem.* 2022;8(1):56. 10.1186/s40900-022-00391-5 36266671 PMC9585787

[ref-101] Kuckertz A Block J : Reviewing systematic literature reviews: ten key questions and criteria for reviewers. *Manag Rev Q.* 2021;71:519–24. 10.1007/s11301-021-00228-7

[ref-102] Land Information New Zealand: Statement of intent 2015–2019.(Wellington: New Zealand Government),2015. Reference Source

[ref-103] Landry C Caust M : The creative bureaucracy and its radical common sense.Stroud: Comedia,2017. Reference Source

[ref-104] Leadbeater C Winhall J : Building better systems a green paper on system innovation.London: Rockwool Foundation,2020. Reference Source

[ref-105] Lederach JP : The moral imagination.Oxford: Oxford University Press,2005. 10.1093/0195174542.001.0001

[ref-106] Leithwood K McCullough C : De-mystifying effective district leadership.OPC Register,2015;17(3):28–31. Reference Source

[ref-107] Lichtenstein B : A matrix of complexity for leadership.In: J. Hazy, J. Goldstein, and B. Lichtenstein (Eds.) *Complex systems leadership theory.*Boston, MA: ISCE Press,2007;285–304.

[ref-108] Lichtenstein B Uhl-Bien M Marion R : Complexity leadership theory: an interactive perspective on leading in complex adaptive systems. *Emergence: Complexity and Organization.* 2006;8(4):2–12. Reference Source

[ref-109] Lichtenstein BB Plowman DA : The leadership of emergence: a complex systems leadership theory of emergence at successive organizational levels. *Leadersh Q.* 2009;20(4):617–630. 10.1016/j.leaqua.2009.04.006

[ref-110] Local Government Association: Shifting the centre of gravity: making place-based, person-centred health and social care a reality.London: LGA,2018. Reference Source

[ref-111] Malby R Mervyn K Boyle TJ : Darzi clinical leadership fellows: an activity theory perspective. *J Health Organ Manag.* 2018;32(6):793–808. 10.1108/JHOM-05-2018-0133 30299225

[ref-112] Mangan C Lawrence-Pietroni C : More rave than waltz -Why the complexity of public service means the end for hero leadership.In: Dickinson, H., Needham, C., Mangan, C. and Sullivan, H. (eds) *Reimagining the future public service workforce.*London: Springer,2019;81–92.

[ref-113] Mangan C Pietroni C : Telling stories and turning up the heat: exploring new approaches to developing public servants.In: Sullivan, H., Dickson, H. and Henderson, H. (eds) *The palgrave handbook of the public servant.*London: Palgrave,2021;1453–70. 10.1007/978-3-030-29980-4_40

[ref-114] Manley K Martin A Jackson C : Using systems thinking to identify workforce enablers for a whole systems approach to urgent and emergency care delivery: a multiple case study. *BMC Health Serv Res.* 2016;16(a): 368. 10.1186/s12913-016-1616-y 27507157 PMC4979146

[ref-115] Marion R Uhl-Bien M : Leadership in complex organizations. *Leadersh Q.* 2001;12(4):389−418. 10.1016/S1048-9843(01)00092-3

[ref-116] Mayanja S Ntayi JM Munene JC : Informational differences and entrepreneurial networking among small and medium enterprises in Uganda. *J Glob Entrepr Res.* 2021;11:563–77. 10.1007/s40497-021-00304-y

[ref-117] Meadows D : Leverage points. Places to intervene in a system.Hartland VT: The Sustainability Institute,1999. Reference Source

[ref-118] Meyers CV : An urban district’s struggle to preserve school turnaround change. *Urban Education.* 2024;59(1):300–329. 10.1177/0042085920966031

[ref-119] Miller R : Systems leadership – enhancing the role of social care.Devon: Research in Practice,2020. Reference Source

[ref-120] Ministry of Transport: Road to zero: summary of submission.Wellington: New Zealand Government,2019. Reference Source

[ref-121] Ministry of Transport: Road to zero annual monitoring report 2021.(Wellington: New Zealand Government),2022. Reference Source

[ref-122] Mintrom M : So you want to be a policy entrepreneur? *Policy Des Pract.* 2019;2(4):307–323. 10.1080/25741292.2019.1675989

[ref-123] Moore J Elliott I Hesselgreaves H : Collaborative leadership in integrated care systems; creating leadership for the common good. *Journal of Change Management.* 2023;23(4):358–373. 10.1080/14697017.2023.2261126

[ref-124] Moore M : Creating public value: strategic management in government.(Cambridge, Massachusetts: Harvard University Press),1995. Reference Source

[ref-125] Mowat JG : Closing the gap’: systems leadership is no leadership at all without a moral compass - a Scottish perspective. *School Leadership & Management.* 2019;39(1):48–75. 10.1080/13632434.2018.1447457

[ref-126] National Leadership Centre: Public service leadership: insights from the research landscape.(London: National Leadership Centre),2020. Reference Source

[ref-127] Nel D Taeihagh A : The soft underbelly of complexity science adoption in policymaking: towards addressing frequently overlooked non-technical challenges. *Policy Sci.* 2024;57:403–436. 10.1007/s11077-024-09531-y

[ref-128] Nguyen LKN Kumar C Bisaro Shah M : Civil servant and expert perspectives on drivers, values, challenges and successes in adopting systems thinking in policy-making. *Systems.* 2023;11(4): 193,1–24. 10.3390/systems11040193

[ref-129] NHS Confederation: The future of systems leadership.(out of date link, refers to 2021 report),2014. Reference Source

[ref-130] NHS England (Sustainable Improvement Team and the Horizons Team): Leading large scale change: a practical guide.(London: NHS England),2018. Reference Source

[ref-131] Nilsen P Ståhl C Roback K : Never the twain shall meet?-a comparison of implementation science and policy implementation research. *Implement Sci.* 2013;8:63. 10.1186/1748-5908-8-63 23758952 PMC3686664

[ref-132] O’Flynn J : Crossing boundaries: the fundamental questions in public management and policy.In: O’Flynn, J., Blackman, D. and Halligan, J. (eds). *Crossing Boundaries in Public Management and Policy. The international experience.*(London: Routledge),2013;11–44. Reference Source

[ref-134] O'Flynn J : From new public management to public value: paradigmatic change and managerial implications. *Australian Journal of Public Administration.* 2007;66(3):353–366. 10.1111/j.1467-8500.2007.00545.x

[ref-133] O’Malley C Petit N Lewthwaite A : Changing food systems: what systems thinking means for designing and implementing international development programmes to catalyse change in food and agricultural systems.(New York: United Nations Development Programme),2022. Reference Source

[ref-135] Onyura B Crann S Freeman R : The state-of-play in physician health systems leadership research: a review of paradoxes in evidence. *Leadersh Health Serv (Bradf Engl).* 2019;32(4):620–643. 10.1108/LHS-03-2019-0017 31612784

[ref-136] Onyx J Leonard RJ : Complex systems leadership in emergent community projects. *Community Dev J.* 2011;46(4):493–510. 10.1093/cdj/bsq041

[ref-137] Osborn RN Hunt JG Jauch LR : Toward a contextual theory of leadership. *The Leadership Quarterly.* 2002;13(6):797−837. 10.1016/S1048-9843(02)00154-6

[ref-138] Osborne S : The new public governance? *Public Management Review.* 2006;8(3):377–387. 10.1080/14719030600853022

[ref-139] Ospina SM : Collective leadership and context in public administration: bridging public leadership research and leadership studies. *Public Adm Rev.* 2017;77(2):275–287. 10.1111/puar.12706

[ref-140] Public Policy Forum, The Canadian Agri-food Policy Institute (CAPI): Canada as an agri-food powerhouse.(Ottowa: Public Policy Forum),2017. Reference Source

[ref-141] Reinertsen JL Bisognano M Pugh MD : Seven leadership leverage points for organization-level improvement in health care (second edition). (Cambridge, Massachusetts: Institute for Healthcare Improvement),2008. Reference Source

[ref-142] Reypens C Lievens A Blazevic V : Hybrid orchestration in multi-stakeholder innovation networks: practices of mobilizing multiple, diverse stakeholders across organizational boundaries. *Organ Stud.* 2021;42(1):61–83. 10.1177/0170840619868268

[ref-143] Rhodes RAW : Understanding Governance.(Buckingham: Open University Press),1997. Reference Source

[ref-144] Rigby J Donaldson Walsh E Boten S : A view from the field: the process of improving equitable systems leadership. *J Educ Adm.* 2019;57(5):484–500. 10.1108/JEA-09-2018-0181

[ref-145] Rissola G Haberleithner J : Place-based innovation ecosystems.(Brussels: Joint Research Centre, European Commission),2020. 10.2760/492676

[ref-146] Rittel HWJ Webber MM : Dilemmas in a general theory of planning. *Policy Sci.* 1973;4(2):155–169. 10.1007/BF01405730

[ref-147] Rose ES Bello-Manga H Boafor T : International collaborative research, systems leadership and education: reflections from academic biomedical researchers in Africa. *Front Educ.* 2024;8: 1217066. 10.3389/feduc.2023.1217066 PMC13309179 42369580

[ref-148] Sandelowski M Barroso J : Handbook for synthesising qualitative research.(New York: Springer),2007. Reference Source

[ref-149] Schroeder J Rowcliffe P : Growing compassionate systems leadership: a toolkit.(Canada: The Human Early Learning Partnership),2019. Reference Source

[ref-150] Senge P : The fifth discipline: the art and practice of the learning organization. (New York: Doubleday),1990. Reference Source

[ref-152] Senge P Hamilton H Kania J : The dawn of system leadership. *Stanf Soc Innov Rev.* 2015;13(1):27–33. 10.48558/yte7-xt62

[ref-151] Senge P Sterman JD : Systems thinking and organizational learning: acting locally and thinking globally in the organization of the future. *Eur J Oper Res.* 1992;59(1):137–150. 10.1016/0377-2217(92)90011-W

[ref-153] Silva A : What is leadership? *Journal of Business Studies Quarterly.* 2016;8(1):1–6. Reference Source

[ref-154] Smith C Neve G Manley O : Future systems leadership scoping project. Clinical leadership. (London: NHS Confederation), 2021. Reference Source

[ref-155] Spillane J : Distributed leadership. (San Francisco, CA: Jossey-Bass),2006. Reference Source

[ref-156] Stabler A Kempton L : Putting systems leadership in its place: the potential of universities. (London: National Leadership Centre), 2020. Reference Source

[ref-157] Stacey R : Ways of thinking about public sector governance.In: R. Stacey and D. Griffin (Eds.) *Complexity and the experience of managing in public sector organizations.*(London: Routledge),2006;15–39.

[ref-158] Swanwick T McKimm J : What is clinical leadership… and why is it important? *Clin Teach.* 2011;8(1):22–26. 10.1111/j.1743-498X.2010.00423.x 21324068

[ref-159] Talley EK Hull RB : Systems thinking for systems leadership: promoting competency development for graduate students in sustainability studies. *Int J Sustain High Educ.* 2023;24(5):1039–1057. 10.1108/IJSHE-11-2021-0489

[ref-160] Taylor C Ruddle N Perry K : Addressing avoidable inequalities: the role of one university in place-based transformational change.In: Sengupta, E., Blessinger, P. and Mahoney, C. (Ed.) *University–Community Partnerships for Promoting Social Responsibility in Higher Education*. (Leeds: Emerald),2020;47–59. 10.1108/S2055-364120200000023004

[ref-161] The Institute for Educational Leadership: Self-assessment tool for Catholic school leaders.(Ontario: The Institute for Educational Leadership),2014. Reference Source

[ref-162] The Institute for Educational Leadership: A comprehensive approach to leadership development. Thames valley district school board. (Ontario: The Institute for Educational Leadership),2016. Reference Source

[ref-163] The Institute for Educational Leadership: French-Language Education in Ontario: a fresh perspective on leadership practices (Executive Summary).(Ontario: The Institute for Educational Leadership),2017. Reference Source

[ref-164] The Institute for Educational Leadership: The institute for education leadership of Ontario – 2018–19 annual Report.(Ontario: The Institute for Educational Leadership),2019. Reference Source

[ref-165] The Institute for Educational Leadership: 2019–20 Annual Report.(Ontario: The Institute for Educational Leadership),2020. Reference Source

[ref-166] The Institute for Educational Leadership: Case study 2: leadership for school improvement. (Ontario: The Institute for Educational Leadership),2021a. Reference Source

[ref-167] The Institute for Educational Leadership: Strengthening your psychological Personal Leadership Resources. (Ontario: The Institute for Educational Leadership),2021b. Reference Source

[ref-168] The King’s Fund: The Future of Leadership and Management in the NHS: no more heroes. (London: The King’s Fund), (A: 27.10.24);2011. Reference Source

[ref-169] The Leadership Centre: The Revolution will be Improvised Part II. (London: The Leadership Centre),2015. Reference Source

[ref-170] The Policy Project: Intentionally building our policy people capability across the system. Wellington: New Zealand Government,2015. Reference Source

[ref-171] Thompson CJ Nelson-Marten P : Clinical nurse specialist education: actualizing the systems leadership competency. *Clin Nurse Spec.* 2011;25(3):133–139. 10.1097/NUR.0b013e318217b5c5 21483244

[ref-172] Timmins N : The practice of system leadership. Being comfortable with chaos. London: The King’s Fund,2015; (A: 27.10.24). Reference Source

[ref-173] Turnbull N Hoppe R : Problematizing ‘wickedness’: a critique of the wicked problems concept, from philosophy to practice. *Policy Soc.* 2019;38(2):315–337. 10.1080/14494035.2018.1488796

[ref-174] Uhl-Bien M Arena M : Complexity leadership. *Organ Dyn.* 2017;46(1):9–20. 10.1016/j.orgdyn.2016.12.001

[ref-175] Uhl-Bien M Marion R McKelvey B : Complexity leadership theory: shifting leadership from the industrial age to the knowledge era. *Leadership Quart.* 2007;18(4):298–318. 10.1016/j.leaqua.2007.04.002

[ref-176] US Department of Transportation: Leading the Way to a Safer Transportation System. Washington DC: US Department of Transportation,2016. Reference Source

[ref-177] Van Dierendonck D : Servant leadership: a review and synthesis. *J Manag.* 2011;37(4):1228–1261. 10.1177/0149206310380462

[ref-178] Van Dyke M : Systems Leadership: exceptional leadership for exceptional times. London: Colebrooke Centre for Evidence and Implementation,2013. Reference Source

[ref-179] Walker A Sillett J Hussain F : Local health systems: relationships not structures. London: LGIU,2022. Reference Source

[ref-180] Waring J Bishop S Black G : Understanding the political skills and behaviours for leading the implementation of health services change: a qualitative interview study. *Int J Health Policy Manag.* 2022;11(11):2686–2697. 10.34172/ijhpm.2022.6564 35297229 PMC9818121

[ref-181] Waring J Bishop S Black G : Navigating the micro-politics of major system change: the implementation of sustainability transformation partnerships in the English health and care system. *J Health Serv Res Policy.* 2023a;28(4):233–243. 10.1177/13558196221142237 36515386 PMC10515458

[ref-182] Waring J Bishop S Clarke J : Becoming active in the micro-politics of healthcare re-organisation: the identity work and political activation of doctors, nurses and managers. *Soc Sci Med.* 2023b;333: 116145. 10.1016/j.socscimed.2023.116145 37572631

[ref-183] Waring J Bishop S Clarke J : Healthcare leadership with political astuteness (HeLPA): a qualitative study of how service leaders understand and mediate the informal ‘power and politics’ of major health system change. *BMC Health Serv Res.* 2018;18(1): 918. 10.1186/s12913-018-3728-z 30509270 PMC6276206

[ref-184] Waterloo Catholic District School Board: Leadership and succession planning framework 2010-2013.(Waterloo: Leadership and Succession Planning Framework 2010-2013). 2011. Reference Source

[ref-185] Welbourn D Warwick R Carnall C : Leadership of whole systems.(London: King’s Fund),2012. Reference Source

[ref-186] Welsh Government: Theme 1: Leadership for a self-improving system Resources 1.(Cardiff: Welsh Government),2016. Reference Source

[ref-187] Wenger-Trayner E Wenger-Trayner B : Systems Convening. A crucial form of leadership for the 21st century.(Sesimbra: Social Learning Lab),2021. Reference Source

[ref-188] West M Eckert R Collins B : Caring to change: how compassionate leadership can simulate innovation in health care.(London: The King’s Fund),2017. Reference Source

[ref-189] Wheatley M : Leadership and the new science.(San Francisco: Berrett-Koehler),1999. Reference Source

[ref-190] Williams HP : Horses preparing superintendent candidates for the leadership Arena. *J Exp Educ.* 2021;44(1):84–96. 10.1177/1053825920966340

[ref-191] Wratten K : Implementing teacher performance and development frameworks - some enabling factors: a review of the international evidence-based literature 2008–18.(Canberra: Parliament of Australia Department of Parliamentary Services),2018. Reference Source

