# Supplementary material for: Systems Leadership: a qualitative systematic review of advice for policymakers
Source: Open Res Eur. 2025 Jan 10;5:6. [Version 1] doi: 10.12688/openreseurope.18982.1 (PMC11862379; doi:10.12688/openreseurope.18982.1)
Supplement: Peer review history

# Reviewer report: Reviewer response for version 1

**DOI:** 10.21956/openreseurope.20542.r50677

**Published:** 2025-04-02

**Authors:** Vesa Koskimaa

**Affiliations:** 1 Tampere University, Tampere, Finland

Thank you for providing me an opportunity to read this well-executed review. The thorough and rigorous analysis that is based on suitable materials and solid interpretation manages to clarify the rather eclectic concept of systems leadership. While doing so, the study details comprehensively the central challenges of the field. The work thus benefits in many ways the students of contemporary political and administrative leadership.

 Inevitably, a work of such scope and magnitude comes with some limitations, especially in regard to conceptual coherence, clarity and relevance (as the authors also acknowledge). But as the limitations largely stem from the challenging task – to disentangle and summarize such a multifaceted and complex concept through several independent studies from various administrative fields and levels – and the analysis itself is indeed very well executed, my reflections should be taken as general notes and ideas rather than concrete suggestions to be applied as such in the possible later iterations of this specific paper.

 First, although I understood the clearly described basic logics of data collection (i.e., on which general criteria and from where the studied articles were sourced from), I found myself missing a bit more detailed description of the motivations and ‘ingredients’ of the source texts. Regarding motivations, I was bit surprised by the seemingly neutral and equal treating of scientific articles and ‘grey papers’ as source material. While scientific articles – the other main category of source texts – can perhaps be approached from such neutral perspective (i.e., as a source of objective administrative advice), at least for a classically trained political scientist like me ‘grey papers’ – the other main category of source texts – raise suspicions of more partial/interest-based motivations (i.e., reflecting organizational ambitions of some specific organizational actors with vested interest in the planned organizational reforms). These could be acknowledged more explicitly.

 Regarding ‘ingredients’, i.e., what the source materials study, the authors perhaps could have provided a bit more detail. While I understand this effort’s broad ambition and scope, level of generality and the limits that come with these choices, I did not fully understand the 'organizationally neutral' treating of papers that concern varying policy sectors and levels of administration. In my opinion the authors could have emphasized a bit more the functional and organizational differences of the specific policy fields and administrative levels which could explain variation in the independent conceptions regarding systems leadership.

 In sum, while I understand the authors’ main  ambition to describe the plethora of conceptions to find potential convergencies, in my opinion the analysis which seems to uncover more heterogeneity than homogeneity would benefit from a bit more explicit recognition of potential political (or interest-based) and scientific sources of differences.

 My second general comment concerns conceptual complexity, which builds on the inclusive and meticulous treating of the source materials that is demanded by the grand research task. The authors are naturally well aware of the issue which stems directly from the source materials, reflecting the reality of the field, and they address it in the paper several times, turning it into a strength.

 The very nicely executed and comprehensive review is also well balanced, as the various positive aspects of systems leadership (or aspects that conform to the general storyline) are countered with a careful inclusion and rigorous analysis of more critical aspects and conceptions. This makes the authors’ portrayal of systems leadership feel fresh and genuinely aimed for purposeful organizational development. It also highlights very well the various challenges of such reforms. 

 At the same time, although the problem indeed results directly from the dispersed views presented in the source materials as was noted above, we should note the associated downside of such comprehensiveness: the inductive open net strategy which considers both academic and more practical advice increases the number of analytic/theoretical viewpoints and categories a lot. While producing a broad and eclectic main concept (which can be an asset in this kind of grand review), the subcategories sometimes also feel rather close to each other, inducing a feeling of repetition occasionally.

 A related challenge concerns the descriptive accuracy of the studied categories. As the various analytical concepts are illustrated with direct quotes from studies that deal with varying policy fields and levels of administration, the exact nature of these actors and activities can remain bit unclear sometimes. For example, if the field and scope of described leadership activities (political, administrative, etc.) is not defined explicitly alongside the illustrative quote, it becomes harder to make sense of the quotes, as the fields entail quite different logics of selection and power dynamics. For example, a casually expressed desire for more dispersed organizational power resources for countering some externally defined ‘wicked problem’ which may seem realistic and pressing in some administrative domain may seem highly unrealistic in some more political domain where the acquisition of, and holding on to, power is a central prerequisite for any meaningful action.  

 While the analytic strategy to include various source texts and theoretical categories is perfectly defendable for this kind of grand review, the strategy also makes the paper quite complex and arduous to process, and it is not always clear how the details serve some more general objective like conceptual development and clarity. As the complexity indeed stems largely from the eclectic descriptions in the source materials which reflect the best understanding of this emerging phenomenon, one could question whether the concept (systems leadership) is still too broad and underspecified for a focused review. An associated challenge, which the authors also raise, is that the existing descriptions of systems leadership sometimes (or maybe even quite often) reminisce some more established theories of network governance.

 For me, the clearest and most substantial contribution of this comprehensive and nicely executed review is exactly this uncovering of the various conceptual and practical tensions that surround abstract concepts like systems leadership that might be relatively easy to draft on paper but which is likely very demanding to apply in practice, especially when the application demands substantial organizational reforms.

Are sufficient details of the methods and analysis provided to allow replication by others?

Yes

Are the rationale for, and objectives of, the Systematic Review clearly stated?

Yes

Is the statistical analysis and its interpretation appropriate?

Yes

Are the conclusions drawn adequately supported by the results presented in the review?

Yes

Reviewer Expertise:

Political science with a special focus on the interplay of political and administrative elites in policymaking.

I confirm that I have read this submission and believe that I have an appropriate level of expertise to confirm that it is of an acceptable scientific standard.

# Reviewer report: Reviewer response for version 1

**DOI:** 10.21956/openreseurope.20542.r49661

**Published:** 2025-02-25

**Authors:** Richard Bolden

**Affiliations:** 1 University of the West of England, Bristol, England, UK

This is a substantial review that draws together a wide body of scholarly and practitioner literature on systems leadership. It focusses specifically on the application of systems leadership in government policymaking and hence may be less relevant/applicable to those seeking to apply systems leadership in other contexts, such as the role of activist groups (e.g. Extinction Rebellion or Occupy) in mobilising social change and/or global partnerships and collaboration (such as the UN Sustainable Development Goals). This is not a problem but could be spelt out more clearly in the abstract, introduction and discussion/conclusion so that readers are clear about the aims and limits of the review.

 Whilst the methodology is well presented, the basis on which articles/outputs were excluded from the review (1,460 at initial screening and 260 at subsequent screening) could be explained more clearly (see Figure 1). It would also be useful to say a bit more about what you mean by a ‘narrative systematic review’ (p6) and to include citations if there was a particular approach that guided your work.

 The findings and analysis section of the review is comprehensive and well presented, although there are places where there are long lists of bullet points, which can be difficult to work through. Where there are repeated bulleted lists (e.g. p16-18) consider how they could be condensed and/or summarised with a connecting paragraph.

 There are a few places where authors are cited who have used/summarised particular ideas rather than original sources – e.g. Edmonstone on the quote from the Leadership Centre (p11), Waring et al. rather than Hartley and colleagues on political astuteness (p19), Talley & Hull rather than Drath et al. on DAC (p19). There are also a few areas where certain references/sources seem to be missing – e.g. Ernst and Chrobot-Mason (and CCL more generally) on boundary spanning leadership (p15) and Jean Hartley and colleagues on political astuteness (p19). Overall, however, these are fairly minor omissions given the scale and rather fragmented nature of the literature covered.

 The discussion provides a useful synthesis of insights, as captured in Table 5. I’m a little unsure, however, what the authors are getting at in the final sentence, which sounds rather dismissive of the potential application of systems leadership in policymaking, and I suggest that this is reviewed/revised if needed. The conclusion provides a good summary of the article, although the list of bullet points could perhaps be condensed/integrated. I also wonder if the authors have any suggestions for future research in this area and/or limitations to the generalisability of findings to other contexts, countries, issues, etc.?

 Overall, this is a useful contribution to the literature on systems leadership that should be of value to academics, educators and practitioners as well as policymakers.

Are sufficient details of the methods and analysis provided to allow replication by others?

Yes

Are the rationale for, and objectives of, the Systematic Review clearly stated?

Yes

Is the statistical analysis and its interpretation appropriate?

Yes

Are the conclusions drawn adequately supported by the results presented in the review?

Yes

Reviewer Expertise:

Leadership

I confirm that I have read this submission and believe that I have an appropriate level of expertise to confirm that it is of an acceptable scientific standard.

# Reviewer report: Reviewer response for version 1

**DOI:** 10.21956/openreseurope.20542.r49656

**Published:** 2025-01-25

**Authors:** Justin Waring

**Affiliations:** 1 University of Loughborough, Loughborough, Leicestershire, UK

I very much enjoyed reading this review and look forward to using it in research and teaching, if it is accepted for indexing. As with the other reviews in this set, it shows greater robustness of method, excellent interpretative skills, and solid recommendations/advice for practitioners and scholars. Below I have some comments and reflections, offered in the spirit of constructive feedback, and I acknowledge that not all are necessarily relevant for this particular review.

 The introduction might locate the concept and trend in a wider context beyond public policy, including the wider literature on leadership and system leadership found in the business and management literature. This could consider related concepts such as distributed leadership and shared leadership (I see these are mentioned later in the results).

 I  very much like the premise of the carrying out these reviews and idea of distilling from the literature advice to inform better policymaking, especially as it does not aim to offer a how-to guide. There is, however, a slight (and probably irrelevant) concern in how this is presented and then becomes translated in ‘what advice do scholars and practitioners offer on system leadership?’

 My thinking is as follows - advice might be generally seen as the giving of insight and recommendations; the advice offered in individual source materials might not always be be advice for policy or practitioners, but rather other scholars or researchers relevant to their broader academic debates; it might not be advice rather argument and analysis; and arguably these people might offer different advice for different audiences, such as theoretical advancement; and more importantly the insights developed through this particular review are the result of the interpretative work and synthesis of the authors. So the framing might be better: what advice can this review offer based upon the work of scholars and practitioners’.

 In terms of methods, did the screening and selection process involve any form of checking or validation with inter-rater validation, e.g Kappa score. If not this migth be a limitation.

 In the first section of the findings, I would like to know more about where the different source materials were located, specifically the types of journals, the disciplinary fields they originated from and the types of conversations they were having - for example there is a difference between the management journal, a health service journal and a political science report in terms of the rationale for the output.

 Looking closer at some of the ideas addressed under the section on ‘what is systems leadership’ I was also left wondering whether a related question(s) might be ‘when’ is systems leadership (or when not) and ‘how’ - meaning that there is something in the literature (implicitly) about that differentiates system leadership also in terms of when it is needed, how it is practices as much as what it involves. This comes through in the following section on wicked problems but I wonder if it goes beyond the problem framing to other factors such as number and diversity of actors involved? But I wonder whether offering a slighting different reading in what of what, when and how might offer additional insight?

 Another interesting line of think, from Keith Grint - is something around the emotional aspect of system leadership - and perhaps leadership more generally? This then made be think about system leadership as an embodied practice is different from other forms of leadership?

 I would encourage greater critical reflection on the idea that systems leadership is more democratic and effective. This section rightly notes that this line stems from the more enthusiastic advocates, but there is limited evidence to these claims and in fact it is entirely plausible that the opposite could be true - in that system leadership works in non-democratic ways through the the more nefarious manipulation of the system.

 The section on the wider debates on leadership is useful but it is also quite dense and covers a vast literature and series of debates but in ways that perhaps does not do justice to the ideas and debates associated with terms like distributed, collective, plural, shared leadership etc. I am not sure that this can be resolved but so maybe a more explicit caveat might help?

 The section that delves into the different requirements is excellent and nicely summarises a complex literature. One dimension that seemed subsumed in this review, but seemed central to our work on political skill, was around self- and positional awareness - i.e. being aware of one’s presentation and reception, and adaptive to the reactions of others; AND being aware of one’s social position relative to others - after all system leadership does not evolve from a blank canvas but emerges from a rich fabric of pre-existing hierarchies and social networks.

 The concluding sections offer a very clear and robust summary of the literature with some clear guidance on what system leadership might involve and how it can be enacted.  One final thought, I was left thinking whether there was a time we might move on from system leadership - what next - how can we move to a more stabilised and less complex environment, or does systems leadership recreate the need for itself - as a type of self-fulfilling prophecy?

 Very minor point, it is stated that the review is focused on policy making but it might also mention policy implementation and service delivery, especially since system leadership spans the formulation and implementation processes.

Are sufficient details of the methods and analysis provided to allow replication by others?

Yes

Are the rationale for, and objectives of, the Systematic Review clearly stated?

Yes

Is the statistical analysis and its interpretation appropriate?

Yes

Are the conclusions drawn adequately supported by the results presented in the review?

Yes

Reviewer Expertise:

Sociology, public administration,

I confirm that I have read this submission and believe that I have an appropriate level of expertise to confirm that it is of an acceptable scientific standard.
